# Meningococcal Genetic Variation Mechanisms Viewed through Comparative Analysis of Serogroup C Strain FAM18

**DOI:** 10.1371/journal.pgen.0030023

**Published:** 2007-02-16

**Authors:** Stephen D Bentley, George S Vernikos, Lori A. S Snyder, Carol Churcher, Claire Arrowsmith, Tracey Chillingworth, Ann Cronin, Paul H Davis, Nancy E Holroyd, Kay Jagels, Mark Maddison, Sharon Moule, Ester Rabbinowitsch, Sarah Sharp, Louise Unwin, Sally Whitehead, Michael A Quail, Mark Achtman, Bart Barrell, Nigel J Saunders, Julian Parkhill

**Affiliations:** 1 Wellcome Trust Sanger Institute, Hinxton, United Kingdom; 2 Bacterial Pathogenesis and Functional Genomics Group, Sir William Dunn School of Pathology, University of Oxford, Oxford, United Kingdom; 3 Molekulare Biologie, Max-Planck Institut für Infektionsbiologie, Berlin, Germany; The Institute for Genomic Research, United States of America

## Abstract

The bacterium *Neisseria meningitidis* is commonly found harmlessly colonising the mucosal surfaces of the human nasopharynx. Occasionally strains can invade host tissues causing septicaemia and meningitis, making the bacterium a major cause of morbidity and mortality in both the developed and developing world. The species is known to be diverse in many ways, as a product of its natural transformability and of a range of recombination and mutation-based systems. Previous work on pathogenic *Neisseria* has identified several mechanisms for the generation of diversity of surface structures, including phase variation based on slippage-like mechanisms and sequence conversion of expressed genes using information from silent loci. Comparison of the genome sequences of two N. meningitidis strains, serogroup B MC58 and serogroup A Z2491, suggested further mechanisms of variation, including C-terminal exchange in specific genes and enhanced localised recombination and variation related to repeat arrays. We have sequenced the genome of N. meningitidis strain FAM18, a representative of the ST-11/ET-37 complex, providing the first genome sequence for the disease-causing serogroup C meningococci; it has 1,976 predicted genes, of which 60 do not have orthologues in the previously sequenced serogroup A or B strains. Through genome comparison with Z2491 and MC58 we have further characterised specific mechanisms of genetic variation in *N. meningitidis,* describing specialised loci for generation of cell surface protein variants and measuring the association between noncoding repeat arrays and sequence variation in flanking genes. Here we provide a detailed view of novel genetic diversification mechanisms in N. meningitidis. Our analysis provides evidence for the hypothesis that the noncoding repeat arrays in neisserial genomes (neisserial intergenic mosaic elements) provide a crucial mechanism for the generation of surface antigen variants. Such variation will have an impact on the interaction with the host tissues, and understanding these mechanisms is important to aid our understanding of the intimate and complex relationship between the human nasopharynx and the meningococcus.

## Author Summary

Human surface tissues, including the skin and gut lining, are host to many different species of bacteria. N. meningitidis is a species of bacteria that is only found in humans where it is able to colonise mucosal surfaces of the nasopharynx (nose and throat). This association is normally harmless and at any one time around 15% of the population are carriers. Some strains of N. meningitidis can cause disease by invading the host tissue leading to septicaemia or meningitis. We aim to gain understanding of the mechanisms by which these bacteria cause disease by studying and comparing genomes from different strains. Here we describe specific genes and associated repetitive DNA sequences that are involved in variation of the bacterial cell surface. The repeat sequences encourage the swapping of genes that code for variant copies of cell surface proteins. The resulting variation of the bacterial cell surface appears to be important in the close interaction between host and bacteria and the potential for disease.

## Introduction

N. meningitidis (the meningococcus) colonizes the nonciliated columnar mucosal cells of the human nasopharynx as a harmless commensal organism and, as such, is carried by five to ten percent of the adult population [1,2]. Some strains are able to cross the mucosa into the bloodstream from where they can cause septicaemia or meningitis and, as a result, are a major cause of disease worldwide [2]. Several genetic loci have been associated with disease [3,4], but for most strains the mechanism of virulence is not well defined. The close interaction with the human host is reflected in enriched diversity and variability at the bacterial cell surface. There are 12 different polysaccharide capsules, which are the basis of serogrouping, some of which are virulence determinants [5–7]. Vaccines targeted to the capsule types most commonly associated with disease have been successful, though capsule switching is a cause of concern [8]. Many meningococcal surface-exposed proteins and carbohydrates are also highly variable, creating a major challenge in the development of a universal meningococcal vaccine [9,10].

Current models of bacterial populations describe a spectrum of structures ranging from clonal, where lineages are derived from a common ancestor and horizontal genetic exchange plays no role, to nonclonal (or panmictic), where rates of horizontal genetic exchange are so high that genetic differences between isolates are effectively randomised and individual genetic lineages are undetectable [11]. Extremes are rare with many bacteria having a semiclonal structure where horizontal exchange is common, but groups of clonally related bacteria exist. Multilocus sequence typing has played a major role in defining bacterial population structure and shows N. meningitidis to have a fundamentally nonclonal population due to the natural competence and high rates of recombination that characterise the species [12–14]. However, multilocus sequence typing is able to resolve N. meningitidis into groups of related sequence types known as clonal complexes, and studies have shown that while there is enormous diversity in the population as a whole there are relatively few lineages associated with the ability to cause disease [15,16]. Most disease causing strains belong to serogroups A, B, or C, but it is clear that membership of one of the hyperinvasive lineages is equally predictive of the ability to cause disease. The genomes we analyse here represent three (of around ten) such disease associated lineages (Table 1), and it is hoped that comparative genomics will help to unravel the paradox of devastating virulence in an organism that relies on asymptomatic carriage and person-to-person transmission for its proliferation.

**Table 1:**
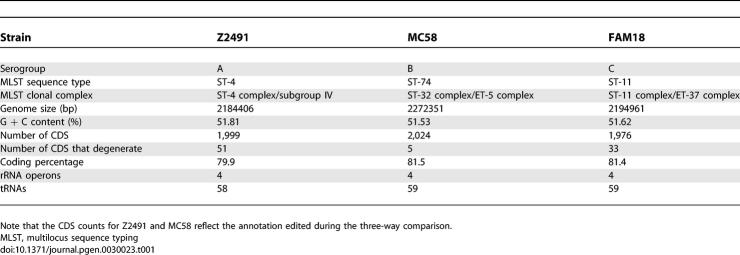
General Features of Three N. meningitidis Genomes

The genome sequences of N. meningitidis strains Z2491 [17] and MC58 [18], which belong to serogroups A and B respectively, have been reported previously and allowed the initial identification of both known and potentially novel mechanisms for variation of surface structures, including the transfer of coding information from silent gene cassettes, phase variation through slippage-like mechanisms, local recombination, and the presence of arrays of short noncoding repeats throughout the chromosome. These repeat arrays were postulated to increase the variability of the associated genes through enhanced recombination with externally acquired DNA [17]. Here we report the genome sequence of N. meningitidis serogroup C strain FAM18, a medically important representative of the ET-37/ST-11 complex, which has been a major cause of meningococcal disease worldwide throughout the last century [19] and, despite low carriage rates, continues to be associated with sporadic outbreaks [20–23]. Strain FAM18 was isolated from the cerebrospinal fluid of a child suffering from meningitis by Dr. Janne Cannon and colleagues in North Carolina in the 1980s and remains capable of infection. The genomes of these three strains have been incorporated into pan-*Neisseria* DNA microarrays and used in three separate comparative genomic hybridisation studies to compare the gene contents of a range of isolates including N. gonorrhoeae strains, invasive and carriage strains of *N. meningitidis,* and commensal species of *Neisseria* [24–26]. These studies highlighted differential acquisition of islands between strains, evidence of horizontal DNA transfer between N. meningitidis and *N. lactamica,* and the potential to define virulence-specific gene sets. Here, we have compared the FAM18 genome data with those of strains Z2491 and MC58, to specifically focus upon local sequence divergence and provide evidence for mechanisms whereby recombination of exogenous DNA with specific chromosomal loci is promoted to generate variation in cell surface antigens.

## Results/Discussion

We sequenced and annotated the genome of N. meningitidis serogroup C strain FAM18 by standard methods and protocols. The addition of the sequence data present in the FAM18 genome sequence to that from the two previously sequenced N. meningitidis genomes (serogroup A strain Z2491 and serogroup B strain MC58) enabled a three-way whole genome comparison between representatives of three disease-associated lineages within this species. Table 1 shows the general features of these strains and their genomes. For convenience we will subsequently refer to the three strains simply as Z2491, MC58, and FAM18.

### Genome Structure

**Figure 1:**
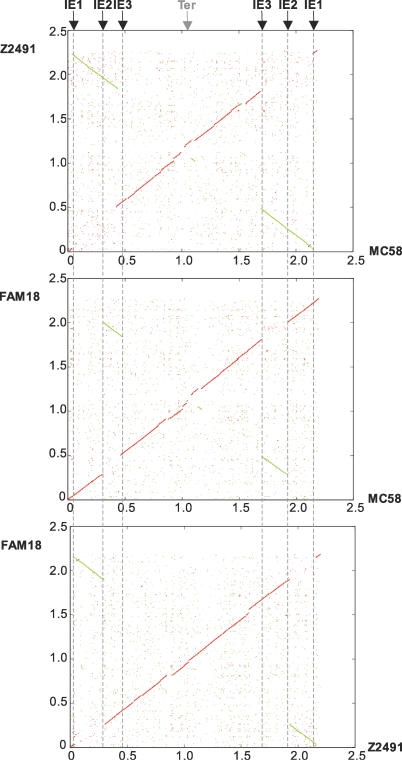
Rearrangements between the Meningococcal Genomes The dotplots were generated using MUMmer version 3.15 (http://mummer.sourceforge.net) and indicate matching sequences with codirectional and reversed regions of synteny shown in red and green, respectively. Genome sequences are aligned to start/finish at the origin of replication with the approximate position of the terminus of replication indicated (Ter) (note this required rotation of the publicly available sequences for Z2491 and MC58, see Materials and Methods). Also shown are the positions of the foci of the three major inversion events (IE1, IE2, and IE3, see text for detail).

The three sequenced N. meningitidis genomes are largely colinear with only three apparent reciprocal inversions around the origin of replication (Figure 1). Each genome appears to represent one of these inversions relative to the other two, suggesting that these inversions may have occurred once in each lineage. However, the foci of the inversions show a high degree of variability relative to the strong synteny across the chromosome, suggesting that they may be subject to frequent additional inter- and intra-genomic recombination.

The inversion event closest to the origin of replication (IE1; 3′-adjacent to NMA0220/NMB0050/NMC0034) seems to be due to recombination between repeat arrays in Z2491. Interestingly, one of the arrays flanks a pilin gene, *pilC2,* while the other is adjacent to genes involved in pilus retraction *(pilTU).* Thus, in MC58 and FAM18, *pilC2* is adjacent to *pilTU,* while in Z2491 they are distant. Neisserial PilC proteins are important components of the type IV pilus machinery involved in adhesion to host cells, promotion of piliation, and transformation competence [27,28]. The PilT protein is essential for pilus retraction [29], and it has been shown that PilC1 regulates PilT-mediated pilus fibre retraction [28]. Although there is no direct published evidence for coregulated transcription of *pilTU* and *pilC2,* it seems plausible that this rearrangement may have an effect on pilus phenotype. The FAM18 pilus regulon also differs from Z2491 and MC58 because of the deletion of much of the *pilE/S* locus and the insertion of the class II pilin-encoding *pilE2* (see below). Further variability at IE1 can be seen with the insertion of a copy of a meningococcal disease associated (MDA) island in the repeat array (see below) directly upstream of *pilC2* in FAM18. The MDA island encodes a filamentous bacteriophage that is secreted via the type IV pilus and is specifically associated with strains that have the potential to cause disease [30].

The second inversion, IE2 (5′-adjacent to NMA2200/ NMB0287/NMC0293), is probably due to recombination between copies of IS1106 in FAM18 and is also associated with the insertion of a locus encoding a putative restriction-modification system in FAM18. Restriction-modification systems coordinate the recognition and destruction of “non-self” DNA from sources lacking the same system and for N. meningitidis have been associated with specific lineages [31].

The third, IE3, is the most complex of the three inversion events and seems to be due to recombination between loci encoding a large repetitive surface protein and its associated secretion system (NMA0688, NMB0497, NMB1779, and NMC0444; see below). These loci appear to encode two-partner, or type V, secretion systems [32] and are similar in sequence and genetic arrangement to those of *Bordetella* species where *fhaC* and *fhaB,* respectively, encode a secretion accessory protein and a filamentous haemagglutinin important in virulence [33]. Z2491 and FAM18 have a single copy of this locus, while MC58 has two copies that are approximately equidistant from the origin of replication and are the foci of the rearrangement. Prior to duplication the locus has also acquired a novel set of two-partner secretion protein genes and an MDA-related prophage. Duplication of the whole locus and subsequent recombination involving another MDA island may have lead to the current genomic arrangement, which would appear to have benefited MC58 with a greater potential variety of surface protein expression.

All three of the reciprocal inversion foci that we have described here seem to affect genetic loci with the potential to modulate interaction with the host and/or other strains of N. meningitidis. Despite frequent inter-strain recombination, N. meningitidis genomes maintain a high level of colinearity, so it may be the case that the rearrangements observed in this three-genome comparison have added significance.

### Three-Way Coding Sequence Comparison

The predicted amino acid sequences of the coding sequences (CDS) from each of the genome annotations were compared by three-way reciprocal Fasta analysis to assess the numbers of orthologous and unique CDS. The latter were defined as CDS where a reciprocal match was not detected in either of the other two translated genome sequences. Visualisation and manual curation of the results of this analysis using the Artemis Comparison Tool revealed limitations of the test. This analysis methodology did not take into account the relative chromosomal position of the genes, so the best matches between genes of the different genomes could be those that are in different chromosomal contexts and, therefore, likely to be paralogues (genes of similar sequence in the same genome) rather than true orthologues. Examples of characteristic features in N. meningitidis that confound the reciprocal match test include CDS within loci encoding variable surface proteins such as adhesins or haemagglutinins. In some cases multiple paralogous loci exist within each genome and may be exchanging DNA by intra- and/or inter-genomic recombination. The result is that syntenic loci (those in the same position) are equally diverged from one another as they are from nonsyntenic loci (see below). For convenience we have designated such genes as “variable” to distinguish them from simple orthologues. Paralogous CDS at nonsyntenic loci are also designated as variable.

Variable genes tend to occur in clusters, and there is a clear correlation between these gene clusters and regions of low % G + C content. Viewed on a whole genome scale, eight of the nine most prominent GC troughs across the genome of FAM18 coincide with variable loci with the one exception being the ribosomal protein operon (NMC0129–NMC0159) (Figure S1). It was observed in Helicobacter pylori that genes that are not universally present across a number of strains, and are therefore likely to be laterally acquired, tend to have a lower than average GC content [34], and a similar bias has been seen in related enteric genomes [35]. It has been suggested that accessory genes (those variably present in different strains within a species) may be subject to different selective pressures to the core genes, and that low % G + C content is one of the results of this difference [36]. It is therefore possible that the low % G + C nature of the variable genes in N. meningitidis may be a consequence of selection for exchange within the species.

In addition, the three meningococcal genome sequences were compared using ACEDB, as described previously [37], to identify unique coding sequences, regardless of their annotation in their respective genomes. The results of these two analysis methodologies were combined and, following manual curation, 240 unique genes were identified; 83 (4.1%) in Z2491, 97 (4.8%) in MC58, and 60 (3.0%) in FAM18. Table 2 summarizes the types and numbers of regions containing unique genes and Table S1 details the individual CDS functional annotations. The majority encode hypothetical proteins of unknown function. This is to be expected, because strain-specific genes are generally poorly studied, and largely do not form part of the common and core metabolic functions that have been most studied, and are most readily identifiable through comparison with other well-studied species and biochemical pathways. There are some unique restriction-modification systems and these would be expected to have an impact on the uptake of DNA from N. menigitidis strains in the same niche; such systems have previously been shown to be associated with different lineages [5,38,39].

**Table 2:**
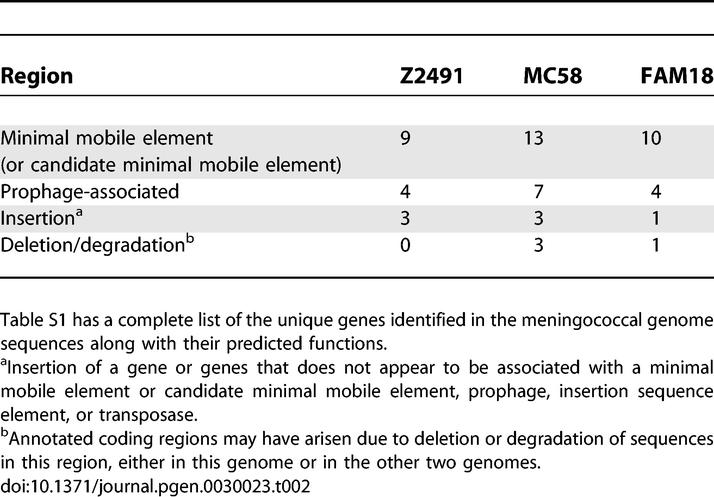
Unique Regions in the Meningococcal Genome Sequences

The majority (39 of 56) of the unique gene clusters contain three or fewer consecutive genes, and 30 of these (68 genes) correspond to known or candidate Minimal Mobile Elements [40] with alternative unique loci present at syntenic locations across the three genomes. With the exception of *dam* in FAM18, all of the unique restriction-modification system genes are within MME*pheST* or MME*rfaDclpA*.

Larger unique regions are often associated with insertion sequence elements (nine of 56 clusters; 53 genes; Table S1) and with a Mu-like prophage (pnm2) present at the same location in all three genomes (between; NMA1280 and NMA1323, NMB1077 and NMB1112, NMC1041 and NMC1056). The IS-associated unique CDS are often small and lie in low % G + C troughs. They are also mostly annotated as “hypothetical proteins” with little information available to allow prediction of the effect of their differential presence. MC58 carries the largest version of prophage pnm2, which includes CDS-encoding cell surface antigens able to induce bacteriocidal antibodies in mice [41]. The presence of unique genes in each genome within these prophage could be due to independent phage insertions, differential gene loss from a larger prophage inserted in a common ancestor, or intergenomic recombination between prophage. Z2491 contains a large unique region of 63 annotated genes (NMA1821–NMA1885), which constitutes a Mu-like prophage (pnm1) shown to be conserved among epidemic serogroup A strains [42], though an association with virulence has not been demonstrated. FAM18 contains a region (NMC0852–NMC0895, IHT-E) that includes genes homologous to lambdoid bacteriophage genes and a transposon carrying a type I secretion system [26].

### Repeat Arrays and Flanking Genes

As with other members of the species, the N. meningitidis FAM18 genome contains many hundreds of repetitive sequence elements ranging from simple sequence repeats associated with phase variable genes (see below), to complete gene cluster duplications (Table 3). DNA uptake sequences (5′-GCCGTCTGAA-3′) are the most abundant repeats and are distributed throughout the genome [43]. Concordant with their % G + C-rich sequence, they are less frequent in low % G + C regions, which often coincide with important genetic loci including those for ribosomal proteins, capsule biosynthesis, pilus biosynthesis, Maf adhesins, prophage, Iga protease, cytolysin transport, and RTX-family exoproteins.

**Table 3:**
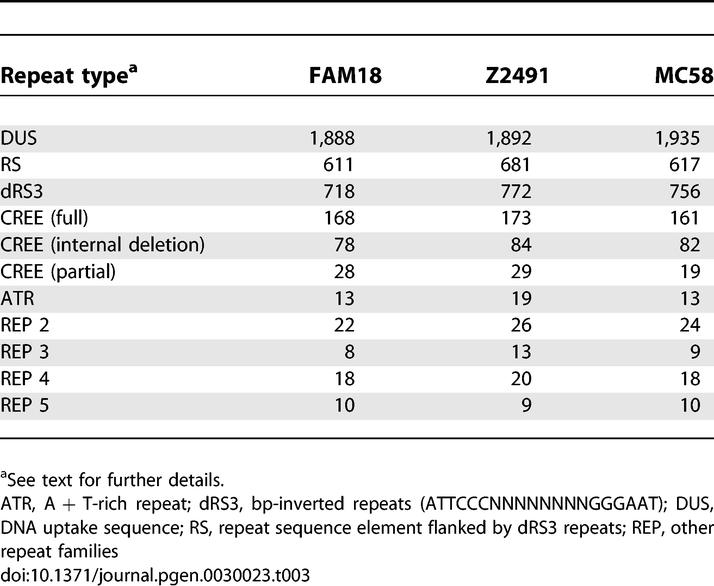
Noncoding Repeat Families Present in FAM18, Z2491, and MC58

The next most abundant repeat types are the “neisserial intergenic mosaic elements” (NIMEs), which consist of 20-bp inverted repeats (ATTCCCNNNNNNNNGGGAAT, dRS3 elements) flanking over 100 families of ∼50–150-bp repeat sequences (RS elements) [17]. Also frequent are the “Correia repeat enclosed elements” (known as CREE or Correia elements), which comprise a conserved repeat sequence (156 bp full length or 51 bp internal deletant) bounded by a 51-bp inverted repeat. CREEs are often located upstream of genes [44], have been shown to affect gene expression [45,46], and may be transposable or mobilisable [47].

The numbers of each major repeat type are comparable in the three complete N. meningitidis genomes (Table 3). Comparison of repeat elements between the three genomes revealed no repeat types unique to one genome though it did identify RS element diversity. For example, repeat sequence clustering analysis for Z2491 and FAM18 showed that of the 611 RS elements in FAM18, there are 80 FAM18-specific versions that group into 27 subfamilies, suggesting novel repeat development, possibly generated by recombination.

**Figure 2:**
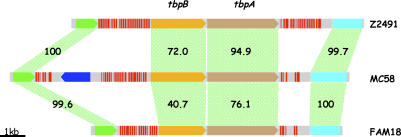
Syntenic Repeat Arrays Showing Variation in Repeat Number and Array Length and *tbpAB* Divergence CDS are shown as arrowed boxes with colours common for orthologues. dRS3 repeat sequences are shown as red lines. Green blocks indicate percentage identity of amino acid sequence between CDS.

NIMEs are often clustered into long arrays of multiple dRS3s separated by different RS elements. These arrays may also contain other repeats such as CREEs and insertion sequence elements, which may be opportunistic insertions. We have previously suggested that these NIME arrays may encourage sequence variation in neighbouring genes by increasing the frequency of recombination with exogenous DNA, and thus exchange of adjacent sequences, either by acting as substrates for homologous recombination, or as targets for a specific recombinase [17]. The chromosomal position of these repeat arrays is generally consistent between the three genomes suggesting that they were initially introduced in a common ancestor. However, comparison of syntenic repeat arrays reveals considerable differences in repeat number and array length, indicating that the arrays themselves are dynamic (Figure 2), as would be expected if they were substrates for recurring recombination.

**Figure 3:**
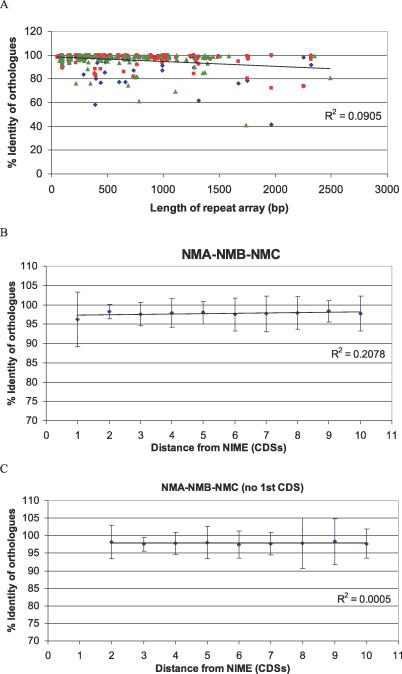
Sequence Divergence in Orthologues Flanking Repeat Arrays**** (A) Plot of repeat array length against flanking orthologue sequence identity for FAM18 versus Z2491 (blue diamond), Z2491 versus MC58 (red square), and FAM18 versus MC58 (green triangle). (B) Plot of distance from array versus orthologue identity for FAM18 versus Z2491, Z2491 versus MC58, and FAM18 versus MC58. (C) The same as (B), ignoring the first CDS.

To study the correlation between repeat arrays and coding sequence divergence, the three pairwise genome comparisons were combined to measure amino acid identities between orthologous CDS. This showed that the average percentage identity between orthologous CDS flanking repeat arrays is significantly (*p*-value = 5.2 × 10^−6^ using a single-tailed *t*-test) lower than the average percentage identity of orthologues not flanking repeat arrays, supporting the hypothesis that the arrays are associated with increased diversity in flanking genes. Despite this strong association, other measures of the diversifying affect of repeat arrays are less clear cut. The relationship between array length and flanking gene diversity is displayed in Figure 3A and shows that for positionally orthologous genes immediately adjacent to repeat arrays there is a tendency for sequence identity to decrease as array length increases and that the same pattern is seen in all three pairwise comparisons. The low combined R^2^ value indicates that the association with repeat length is not strong. However, a strong correlation with array length may be obscured since array size is dynamic (Figure 2) and may itself change rapidly.

We further analysed the orthologue sequence identities to test whether the diversifying affect of the repeat arrays could be detected beyond the immediate adjacent CDS. Figure 3B plots the orthologue identities for the CDS within ten genes of an array and shows a weak association between othologue diversity and distance from the array. Figure 3C shows that this effect is almost entirely due to the diversity seen in the CDS immediately adjacent to arrays. The distance to which the diversifying effect of the arrays extends should be limited by the length of DNA fragment that can be recombined through a double crossover event where one crossover is within the repeat array. A study focused on allele replacement of the *tbpB* gene (Figure 2) encoding an immunogenic cell surface protein in N. meningitidis found that the recombining DNA fragments ranged from 1.5 to 9.9 kb with a median size of 5.1 kb [48]. This size range would suggest that recombination fragments could extend several CDS away from a repeat array, although the effect of this is not seen in our comparisons. Interestingly, of the 19 fragments mapped to *tbpB* allele replacements, 13 had at least one end point within or very close to a flanking NIME repeat array [48], so while immune selection may be driving the retention of variants, the majority of the required recombination events are associated with NIME repeat arrays.

**Figure 4:**
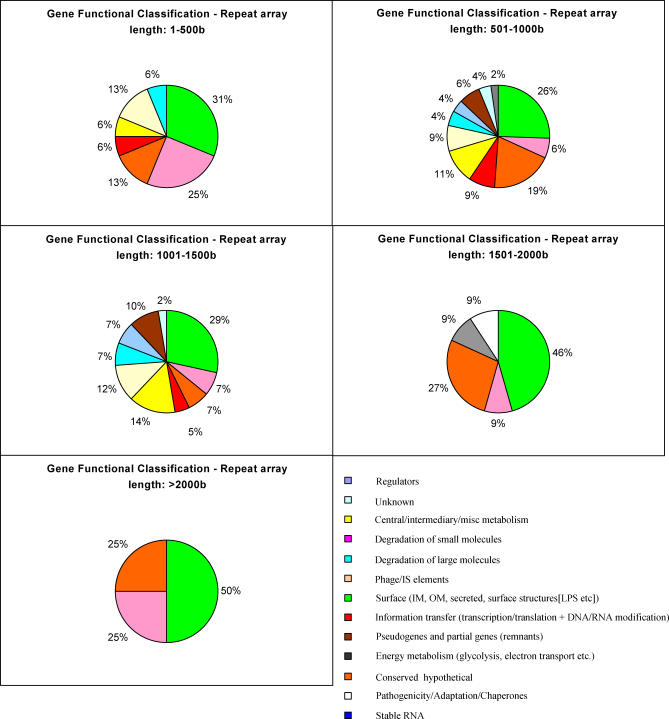
Pie Charts Showing Association between Repeat Arrays and Surface Proteins in FAM18 Chart sectors are coloured according to gene function (see colour key).

Based on the above findings, we hypothesise that the relative positions of CDS and repeat arrays are under selective pressure such that genes where increased variation is beneficial are more likely to be associated with arrays. The repeat arrays serve to promote recombination with exogenously acquired DNA, increasing the rate of gene exchange at the adjacent loci. Although this does not directly cause increased variation in these genes, it should enhance the exchange of variants, and therefore increase the apparent rate of variation. This correlates with the pattern seen in Figure 3A, where some genes are highly variable in some comparisons but not in others—exchange is a stochastic process—sometimes a highly variant gene will have been acquired, and sometimes a gene more similar to the comparators used here. Clearly any exchange will only be fixed if it is selected for, and this may explain why variation from the array is not retained. In support of this hypothesis, there is a clear association between repeat arrays and CDS encoding cell surface associated proteins where increased sequence variation may be an advantage in host interactions (Figure 4; Table S2). At any given repeat array size range, the majority of flanking genes code for cell surface or exported proteins. Moreover, the proportion of this class of genes increases as array size increases. A clear example of the diversifying effect of repeat arrays is the *tbpAB* locus (Figure 2), which lies at a syntenic position in all three genomes flanked by large repeat arrays. The dynamic nature of the repeat arrays is reflected in a wide variation in array size and content. The *tbpA* and *tbpB* DNA sequence identities (74.1%–94.9% and 50.8%–77.8%, respectively) are far below the typical genome figure of >90% and the flanking gene identities of 96.1%–98.5%. Clearly, sequence divergence is focused on *tbpAB* leaving the surrounding genes largely unaffected further implying that selection for advantageous variation is operating. Analogous arrangements can be seen for the *lbpAB* locus, encoding a lactoferrin-binding protein and the *porA* locus, encoding a major outer membrane protein, and the *pilC1/pilC2* loci.

Another consideration is that there may be a reciprocal relationship between the genes undergoing repeated recombination to generate antigenically variable mosaics and the flanking repeats. Since there is little or no selective pressure for accurate recombination within the flanking repeat regions, the recombination within these regions is likely to be more “error prone” than that within the coding regions. So, the recombination of these genes may serve to create variation and growth of the flanking repeats, which in turn may favour further recombination within the adjacent coding regions.

### NIME Array Structure

**Figure 5:**
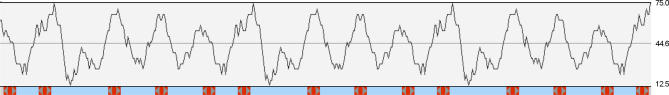
NIME Array Percentage G + C Content Profile for an Array Located at 1283600–1284640 bp in the FAM18 Genome The % G + C window size is 24 bases. Maximum, minimum, and median are also shown. Red blocks represent dRS3 with inverted repeats indicated by grey triangles. Blue blocks represent RS elements.

The NIME arrays themselves display a striking and regular wave profile for % G + C content with troughs corresponding to RS elements and peaks corresponding to dRS3. Figure 5 shows one example but the profile is apparent for all large regular NIME arrays in the genome. The profile is sometimes less obvious in smaller arrays or less regular arrays containing other repeat types such as CREEs, but the % G + C profile is often even detectable for isolated dRS3-RS-dRS3 units. The profile does not appear to be simply due to the high % G + C nature of the dRS3s, but is rather a combination with the lower % G + C RS elements. Average % G + C values for FAM18 are 55.78% for dRS3s, 45.18% for RS elements, and 51.62% for the whole chromosome. Closer analysis shows considerable internal variation for the NIME subunits with dRS3 % G + C ranging from 35% to 70% and RS elements ranging from 28% to 76%. The large overlap between these ranges implies that to maintain the wave profile the dRS3 and RS element sequences are somehow interdependent. Since dRS3s are only 20 bp long and have a conserved 6-bp terminal inverted repeat, their % G + C variation is limited to the central eight bases. RS elements are essentially defined as regions flanked by dRSs, so may not necessarily be functional units and could be largely inert, or have a structural role. Under this definition, RS elements vary in size from 19 to 214 bp and form multiple sequence families some of which tend to have short (5-bp) terminal inverted repeats. Within RS elements % G + C content is often “patterned” with either a central low % G + C trough or a side-to-side slope. Maintenance of the % G + C wave profile in NIME arrays suggests that the structure may be related to function, potentially participating in, or promoting, recombinational events.

We hypothesise that dRS3 sequences within NIME arrays are binding sites for a site-specific recombinase that enhances recombination between these sequences and exogenously acquired DNA containing other dRS3 elements, thereby promoting variation at a number of genes associated with NIME arrays. If the dRS3-mediated recombination formed an initial cross-over event, then the insertion of linear DNA could be completed by RecA-mediated homologous recombination in the adjacent sequences, ensuring replacement with similar genes. Alternatively, pairs of arrays surrounding genes could both participate in dRS3-mediated recombination, or array-flanked genes on acquired DNA could be inserted into chromosomal arrays. Continued recombination between chromosomal and acquired dRS3 elements, with the functionally selected consequence of exchanging adjacent genes, could have the effect of building up repeat arrays containing “spacer” regions (the RS elements) with specific physical or conformational properties.

Bille et al. [30] and Kawai et al. [49] have recently described a type of neisserial filamentous prophage whose presence in meningococcal genomes is associated with the ability to invade host tissues. They have also showed that these bacteriophage integrate into dRS3 repeats by the action of a phage-encoded transposase/recombinase. This protein is therefore a plausible candidate for the specific recombinase predicted by our hypothesis. This phage is a member of a larger family of neisserial phage, and it is therefore reasonable to suppose that this recombinase has been present in the neisserial genome for some time.

### Silent Gene Cassette–Mediated Variation

N. meningitidis genomes contain several loci where transcriptionally silent gene cassettes can be used as sources of variation for expressed surface structures and proteins. Comparison of such variable loci from different strains reveals detail of different genetic arrangements and may be useful for understanding the mechanisms for generation of variants. The best-described example is the pilin-encoding *pilE/S* system where the expressed pilin (PilE) can be altered by incorporation into the *pilE* CDS of DNA from 5′-adjacent promoter-less *pilS* genes [50]. Much of the *pilE/S* locus has been deleted in FAM18, which is associated with the previously recognized insertion, and conversion to the sole expression, of a class II pilin-encoding gene *(pilE2)* elsewhere on the chromosome (which is not present in Z2491 and MC58) [51]. Variation of the *pilE* gene using *pilS* sequences has been extensively studied [50,52,53], the efficiency of which has been shown in N. gonorrhoeae to involve a short DNA sequence (the Sma/Cla repeat) located downstream of the *pilE* gene. In N. meningitidis the silent *pilS* loci are embedded within NIME arrays, and it is possible that the specific dRS3-mediated recombination postulated above may contribute to generating silent variation within *pilS* sequences.

A different mechanism of variation appears to exist for several loci encoding putative haemagluttinins *(fhaB)* and adhesins *(mafB)*. Downstream of these genes are what appear to be silent cassettes encoding alternative C termini for the encoded proteins. These cassettes contain short repeats that are identical to sequences only present within the upstream genes. We have previously suggested that these repeats could be the substrates for direct recombination, replacing the 5′ end of the gene [17]. The three-way comparison provides more evidence in support of this view, including examples where the C terminus of one of the expressed genes in one genome is identical to a silent cassette in the same locus in another genome.

**Figure 6:**
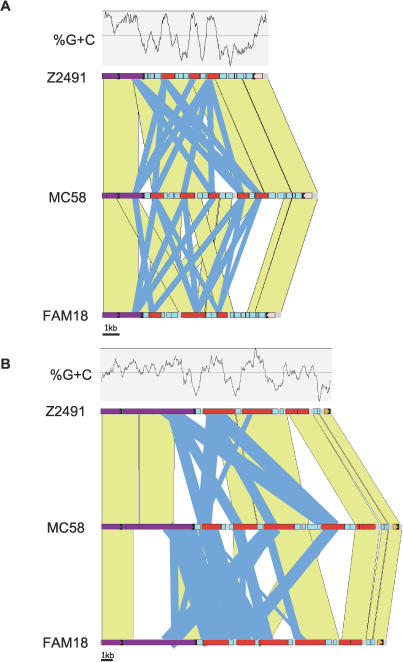
Silent Gene Cassette–Mediated Variation (A) *maf1* locus (NMA0305–0286, NMB2104–2127, NMC2083–2102). (B) *fha* loci (NMA0687–0698, NMB0496–0521, NMC0443–0459). Purple and red boxes indicate the coding sequences and corresponding downstream alternative C termini-encoding silent sequences, respectively. Pale blue boxes represent intervening annotated coding sequences. Pink and orange boxes represent transposase and hypothetical protein encoding, flanking CDS, respectively. Coloured blocks between gene clusters represent regions of sequence similarity; green blocks indicate large syntenic regions of similarity, and blue blocks represent shorter internal repeat sequences, which may facilitate recombination between loci.

The *maf* loci are generally comprised of tandem *mafA* and *mafB* genes, both of which are thought to encode adhesins, followed by a number of putative silent cassettes, and many genes of unknown function (Figure 6A). Each of the three N. meningitidis genomes has three *maf* loci designated here as *maf1*, *maf2,* and *maf3* (based on their order relative to the origin of replication in the FAM18 genome sequence). The loci are at syntenic positions but occur in a different order within each chromosome due to the chromosomal inversion events described above (Figure 1). For all three genomes the *maf2* and *maf3* loci start with a *mafA* gene, which is absent from *maf1*. However, *maf1* from MC58 *(maf1_MC58)* and FAM18 *(maf1_FAM18)* have a truncated *mafA* mid way through the locus, and *maf1_Z2491* has been largely deleted. There is also a possible *mafA* remnant in the middle of *maf3_FAM18*. Clearly there is considerable variation encoded within the *maf* loci, which would be expected to manifest as variations in adhesin structure at the cell surface.

Although all three *maf* loci have a similar structure and appear to be encoding a similar product, at the sequence level *maf1* and *maf2* are more similar to each other, with *maf3* having only localised similarity. DNA identities between *maf1* and *maf2 mafA* genes are high (>97%), but their identities to *maf3 mafA* genes are much lower (∼65%). Identities between *maf3 mafA* genes are greater than 98%. An analogous situation exists for the *mafB* sequences. The encoded *maf3* MafAs have an N-terminal extension relative to the others but all appear to have an intact signal sequence and are likely to be exported.

At 41.2%, the average % G + C content of *maf* loci is markedly lower than the genome average of 51.5%. Furthermore, there is a distinct profile of % G + C content across *maf* loci. Generally *mafA* and *mafB* CDS, including the downstream alternative CDS, correspond to % G + C peaks with some *mafA* CDS as high as 59% GC. Although the intervening % G + C troughs have been annotated with potential CDS, they have no similarity to genes in the database, and their role is unclear. It is notable that they do not contain any of the repeats associated with repeat arrays.

FAM18 and Z2491 have single syntenic *fha* loci, while MC58 has two that are associated with a genome inversion event (IE3) as described above (Figure 1). These loci are analogous to the *fha* loci in Bordetella pertussis with an upstream *gene (fhaC)* encoding a two-partner secretion system outer membrane transporter [54], though the B. pertussis loci do not have downstream silent cassettes. The *N. meningitidis fha* loci have similar structure to the *maf* loci with silent cassettes directly downstream from a gene encoding a large low complexity surface protein and a distinct % G + C profile (Figure 6B). The size of the repeats shared by the silent cassettes and the functional gene is comparable for both *fha* and *maf*. DNA identities for *N. meningitidis fhaC* and the 5′ portion of *fhaB* are greater than 98%. The degree of similarity between *fhaB* genes is variable with the two copies in MC58 having a high level of identity over almost the full length, while for Z2491 and FAM18 gene identity is largely confined to the 5′ end with the remainder either unique or having similarity in the downstream “silent” region.

The *maf* and *fha* loci show considerable potential for generation of multiple versions of the expressed coding sequence and, together with surface structures such as pilus, capsule, and other surface proteins, are likely to be major contributors to cell surface diversity. The presence of multiple syntenic *maf* loci is striking and suggests an important role.

### Phase Variable Genes

Previously, a number of potential phase variable genes have been identified based on the presence of potentially slippage-prone short repetitive sequences, and these lists have been progressively refined, through analysis of neisserial genome sequences, first in N. meningitidis strains MC58 [18] and Z2491 [17] and then in comparative studies using both of these and N. gonorrhoeae strain FA1090 [37], and subsequently in a study of the commonly used experimental N. gonorrhoeae strains [55] and a partial study of N. meningitidis [56]*.* Based upon these studies, and those published by others on specific genes, and a four-way comparison using the N. meningitidis FAM18 genome sequence, a revised and updated phase variable gene list is presented here (Table S3). There are now 24 known phase variable genes in the Neisseria spp. and a further 25 strong candidates, counting members of established gene families such as Opa proteins only once. Over half of these encode surface proteins, enzymes that modify surface proteins, or are LPS biosynthesis proteins. This mechanism therefore has a vast capacity to vary the surface-exposed structures and epitopes of N. meningitidis.

### Concluding Remarks

Based upon the comparisons of the three meningococcal genomes, we further characterized a number of known and putative mechanisms for the generation of diversity within and between strains of this highly adaptable and variable species. Many of these mechanisms involve random variation, which is locally increased due to the presence of repeats, either generating local instability or serving as substrates for homologous recombination, resulting in altered expression of specific genes (phase variation) or generating allelic diversity within particular surface proteins (*pilE, mafB*/*fhaB* families, NIME array-associated genes)

While phase variation through homopolymeric tracts has been noted in several other genera, the NIME arrays and cassette-mediated variation described here seem to be specific to *Neisseria* and may be important characterising features of the genus. NIME arrays are found at syntenic positions in the genomes of N. gonorrhoeae and N. lactamica (http://www.sanger.ac.uk/Projects/N_lactamica) but have not been observed in non-neisserial genomes. Although two-partner secretion systems analogous to the *fha* locus are found in other bacterial genomes [33], they only include the two essential components and lack the silent cassettes that enhance variability in N. meningitidis. Notably, the N. gonorrhoeae and N. lactamica genomes both have three *maf* loci syntenic with those in N. meningitidis, N. gonorrhoeae has two extra *maf* loci, and neither have *fha* loci. These genomic differences will affect the cell surface and may relate to niche differences and interactions with the host.

Variation of the bacterial cell surface is a common theme in host–pathogen interactions and appears to be important for colonisation of new niches and avoidance of the immune system. Such variation may be even more important for commensal organisms, such as *Neisseria*, that remain associated with their hosts for long periods. The genome of FAM18, and its comparison with MC58 and Z2491, highlights N. meningitidis as a paradigm of genomic variability linking a combination of DNA uptake and recombination, minimal mobile elements, intergenic repeat arrays, and phase variation to generate and maintain phenotypic diversity focused at the cell surface.

## Materials and Methods

### Genome sequencing.

N. meningitidis strain FAM18 genomic DNA was prepared as previously described [57]. An approximately 8×-shotgun sequence was produced from a total of 68,352 end-sequences from pUC clones with 1.4–2.0kb inserts using the Big Dye Terminator Cycle Sequencing kit from Applied Biosystems (http://www.appliedbiosystems.com). Reactions were run on Applied Biosystems 3700 sequencers. An approximately 1× coverage was produced from 1,152 end sequences from 10–20 kb inserts cloned into pBACe3.6 and used to scaffold contigs and bridge repeat sequences. The sequence was finished to standard criteria [17]. Sequence assembly, visualisation, and finishing were performed using PHRAP (P. Green, unpublished data; http://www.phrap.org) and Gap4 [58].

### Annotation and genome comparison.

Putative orthologues were identified by reciprocal-best-match FASTA searches between the meningococcal strains Z2491, MC58, and FAM18 protein sequences with cutoffs of 80% sequence length and 30% identity. The orthologue list was manually curated and annotation was transferred for orthologues common to strains Z2491 and FAM18. All other genes were annotated using standard criteria [17], and the complete genome annotation was then manually curated in Artemis [59]. The strain Z2491 EMBL entry has also been resubmitted to reflect the annotation updates generated during this study and the rotation of the sequence to place the origin of replication at the start. Genome comparisons were visualised using the Artemis Comparison Tool [60]. Repeats were defined and annotated using a combination of BLAST [61] and HMMer [62].

In an independent analysis, the complete genome sequences of N. meningitidis strains FAM18, MC58, and Z2491 and N. gonorrhoeae strain FA1090 were analysed using ACEDB (R. Durbin, J.T. Thierry-Mieg, unpublished data, http://www.acedb.org) as described previously [37,55,63,64]. Perfect sequence repeats characteristic of phase variable genes were identified using ARRAYFINDER [65]. Repeats, the annotations from all four neisserial genome sequences, and other sequence features were displayed in their sequence context within ACEDB. Analysis of the potential for simple sequence repeats to generate transcriptional or translational phase variation was determined through analysis of the repeat in the sequence context, as has been done previously [37,55,63,64]. Unique genes were identified as those for which no homology was displayed, the display parameters within ACEDB being set to 1e−50 for DNA identity, and 1e−4 for amino acid similarity. In cases of large paralogous gene families, genes that displayed low homology to only a portion of the gene with an annotated feature from another genome sequence were considered in their wider chromosomal context to determine if the allele is unique or divergent. The results of these independent analyses were combined and curated.

## Supporting Information

Figure S1 Functions Associated with Percentage G + C Troughs across the FAM18 Genome (59 KB PDF)

Table S1 Genes Unique to Each of the Three N. meningitidis Genomes (298 KB DOC)

Table S2 Repeat Arrays and Functional Annotation of Flanking Genes in FAM18 (75 KB DOC)

Table S3 Phase Variable Genes of the Neisseria spp. (221 KB DOC)

### Accession Numbers

The EMBL Nucleotide Sequence Database (http://www.ebi.ac.uk/embl) accession numbers for the genomes discussed in this paper are N. gonorrhoeae (AE004969), N. meningitidis strain FAM18 (AM421808), and N. meningitidis strain Z2491 (AL157959).

## Abbreviations

CDS: coding sequences
CREE: Correia repeat enclosed element
MDA: meningococcal disease associated
NIME: neisserial intergenic mosaic element
RS element: repeat sequence element

## References

[pgen-0030023-b001] Stephens DS, Hoffman LH, McGee ZA (1983). Interaction of Neisseria meningitidis with human nasopharyngeal mucosa: Attachment and entry into columnar epithelial cells. J Infect Dis.

[pgen-0030023-b002] Rosenstein NE, Perkins BA, Stephens DS, Popovic T, Hughes JM (2001). Meningococcal disease. N Engl J Med.

[pgen-0030023-b003] Tzeng YL, Stephens DS (2000). Epidemiology and pathogenesis of Neisseria meningitidis. Microbes Infect.

[pgen-0030023-b004] Snyder LA, Davies JK, Ryan CS, Saunders NJ (2005). Comparative overview of the genomic and genetic differences between the pathogenic *Neisseria* strains and species. Plasmid.

[pgen-0030023-b005] Claus H, Stoevesandt J, Frosch M, Vogel U (2001). Genetic isolation of meningococci of the electrophoretic type 37 complex. J Bacteriol.

[pgen-0030023-b006] Read RC, Zimmerli S, Broaddus C, Sanan DA, Stephens DS (1996). The (alpha2–>8)-linked polysialic acid capsule of group B Neisseria meningitidis modifies multiple steps during interaction with human macrophages. Infect Immun.

[pgen-0030023-b007] Virji M, Makepeace K, Peak IR, Ferguson DJ, Moxon ER (1996). Pathogenic mechanisms of Neisseria meningitidis. Ann N Y Acad Sci.

[pgen-0030023-b008] Swartley JS, Marfin AA, Edupuganti S, Liu LJ, Cieslak P (1997). Capsule switching of Neisseria meningitidis. Proc Natl Acad Sci U S A.

[pgen-0030023-b009] Girard MP, Preziosi MP, Aguado MT, Kieny MP (2006). A review of vaccine research and development: Meningococcal disease. Vaccine.

[pgen-0030023-b010] Urwin R, Russell JE, Thompson EA, Holmes EC, Feavers IM (2004). Distribution of surface protein variants among hyperinvasive meningococci: Implications for vaccine design. Infect Immun.

[pgen-0030023-b011] Spratt BG, Maiden MC (1999). Bacterial population genetics, evolution and epidemiology. Philos Trans R Soc Lond B Biol Sci.

[pgen-0030023-b012] Maiden MC (2006). Multilocus sequence typing of bacteria. Annu Rev Microbiol.

[pgen-0030023-b013] Feil EJ, Maiden MC, Achtman M, Spratt BG (1999). The relative contributions of recombination and mutation to the divergence of clones of Neisseria meningitidis. Mol Biol Evol.

[pgen-0030023-b014] Holmes EC, Urwin R, Maiden MC (1999). The influence of recombination on the population structure and evolution of the human pathogen Neisseria meningitidis. Mol Biol Evol.

[pgen-0030023-b015] Caugant DA (1998). Population genetics and molecular epidemiology of Neisseria meningitidis. Apmis.

[pgen-0030023-b016] Yazdankhah SP, Kriz P, Tzanakaki G, Kremastinou J, Kalmusova J (2004). Distribution of serogroups and genotypes among disease-associated and carried isolates of Neisseria meningitidis from the Czech Republic, Greece, and Norway. J Clin Microbiol.

[pgen-0030023-b017] Parkhill J, Achtman M, James KD, Bentley SD, Churcher C (2000). Complete DNA sequence of a serogroup A strain of Neisseria meningitidis Z2491. Nature.

[pgen-0030023-b018] Tettelin H, Saunders NJ, Heidelberg J, Jeffries AC, Nelson KE (2000). Complete genome sequence of Neisseria meningitidis serogroup B strain MC58. Science.

[pgen-0030023-b019] Wang JF, Caugant DA, Morelli G, Koumare B, Achtman M (1993). Antigenic and epidemiologic properties of the ET-37 complex of Neisseria meningitidis. J Infect Dis.

[pgen-0030023-b020] Nicolas P, Norheim G, Garnotel E, Djibo S, Caugant DA (2005). Molecular epidemiology of Neisseria meningitidis isolated in the African Meningitis Belt between 1988 and 2003 shows dominance of sequence type 5 (ST-5) and ST-11 complexes. J Clin Microbiol.

[pgen-0030023-b021] Nicolas P, Djibo S, Moussa A, Tenebray B, Boisier P (2005). Molecular epidemiology of meningococci isolated in Niger in 2003 shows serogroup A sequence type (ST)-7 and serogroup W135 ST-11 or ST-2881 strains. J Clin Microbiol.

[pgen-0030023-b022] Traore Y, Njanpop-Lafourcade BM, Adjogble KL, Lourd M, Yaro S (2006). The rise and fall of epidemic Neisseria meningitidis serogroup W135 meningitis in Burkina Faso, 2002–2005. Clin Infect Dis.

[pgen-0030023-b023] Mayer LW, Reeves MW, Al-Hamdan N, Sacchi CT, Taha MK (2002). Outbreak of W135 meningococcal disease in 2000: Not emergence of a new W135 strain but clonal expansion within the electophoretic type-37 complex. J Infect Dis.

[pgen-0030023-b024] Stabler RA, Marsden GL, Witney AA, Li Y, Bentley SD (2005). Identification of pathogen-specific genes through microarray analysis of pathogenic and commensal *Neisseria* species. Microbiology.

[pgen-0030023-b025] Snyder LA, Saunders NJ (2006). The majority of genes in the pathogenic *Neisseria* species are present in non-pathogenic Neisseria lactamica, including those designated as ‘virulence genes'. BMC Genomics.

[pgen-0030023-b026] Dunning Hotopp JC, Grifantini R, Kumar N, Tzeng YL, Fouts D (2006). Comparative genomics of Neisseria meningitidis: Core genome, islands of horizontal transfer and pathogen specific genes. Microbiology.

[pgen-0030023-b027] Nassif X, Beretti JL, Lowy J, Stenberg P, O'Gaora P (1994). Roles of pilin and PilC in adhesion of Neisseria meningitidis to human epithelial and endothelial cells. Proc Natl Acad Sci U S A.

[pgen-0030023-b028] Morand PC, Bille E, Morelle S, Eugene E, Beretti JL (2004). Type IV pilus retraction in pathogenic *Neisseria* is regulated by the PilC proteins. Embo J.

[pgen-0030023-b029] Merz AJ, So M, Sheetz MP (2000). Pilus retraction powers bacterial twitching motility. Nature.

[pgen-0030023-b030] Bille E, Zahar JR, Perrin A, Morelle S, Kriz P (2005). A chromosomally integrated bacteriophage in invasive meningococci. J Exp Med.

[pgen-0030023-b031] Bart A, Pannekoek Y, Dankert J, van der Ende A (2001). *NmeSI* restriction-modification system identified by representational difference analysis of a hypervirulent Neisseria meningitidis strain. Infect Immun.

[pgen-0030023-b032] van Ulsen P, Tommassen J (2006). Protein secretion and secreted proteins in pathogenic Neisseriaceae. FEMS Microbiol Rev.

[pgen-0030023-b033] Jacob-Dubuisson F, Kehoe B, Willery E, Reveneau N, Locht C (2000). Molecular characterization of Bordetella bronchiseptica filamentous haemagglutinin and its secretion machinery. Microbiology.

[pgen-0030023-b034] Gressmann H, Linz B, Ghai R, Pleissner KP, Schlapbach R (2005). Gain and loss of multiple genes during the evolution of Helicobacter pylori. PLoS Genet.

[pgen-0030023-b035] Daubin V, Ochman H (2004). Bacterial genomes as new gene homes: The genealogy of ORFans in E. coli. Genome Res.

[pgen-0030023-b036] Young JP, Crossman LC, Johnston AW, Thomson NR, Ghazoui ZF (2006). The genome of Rhizobium leguminosarum has recognizable core and accessory components. Genome Biol.

[pgen-0030023-b037] Snyder LA, Butcher SA, Saunders NJ (2001). Comparative whole-genome analyses reveal over 100 putative phase-variable genes in the pathogenic Neisseria spp. Microbiology.

[pgen-0030023-b038] Davies JK (1989). DNA restriction and modification systems in Neisseria gonorrhoeae. Clin Microbiol Rev.

[pgen-0030023-b039] Claus H, Friedrich A, Frosch M, Vogel U (2000). Differential distribution of novel restriction-modification systems in clonal lineages of Neisseria meningitidis. J Bacteriol.

[pgen-0030023-b040] Saunders NJ, Snyder LA (2002). The minimal mobile element. Microbiology.

[pgen-0030023-b041] Masignani V, Giuliani MM, Tettelin H, Comanducci M, Rappuoli R (2001). Mu-like Prophage in serogroup B Neisseria meningitidis coding for surface-exposed antigens. Infect Immun.

[pgen-0030023-b042] Klee SR, Nassif X, Kusecek B, Merker P, Beretti JL (2000). Molecular and biological analysis of eight genetic islands that distinguish Neisseria meningitidis from the closely related pathogen Neisseria gonorrhoeae. Infect Immun.

[pgen-0030023-b043] Goodman SD, Scocca JJ (1988). Identification and arrangement of the DNA sequence recognized in specific transformation of Neisseria gonorrhoeae. Proc Natl Acad Sci U S A.

[pgen-0030023-b044] Liu SV, Saunders NJ, Jeffries A, Rest RF (2002). Genome analysis and strain comparison of correia repeats and correia repeat-enclosed elements in pathogenic *Neisseria*. J Bacteriol.

[pgen-0030023-b045] Packiam M, Shell DM, Liu SV, Liu YB, McGee DJ (2006). Differential expression and transcriptional analysis of the alpha-2,3-sialyltransferase gene in pathogenic Neisseria spp. Infect Immun.

[pgen-0030023-b046] De Gregorio E, Abrescia C, Carlomagno MS, Di Nocera PP (2002). The abundant class of nemis repeats provides RNA substrates for ribonuclease III in *Neisseriae*. Biochim Biophys Acta.

[pgen-0030023-b047] Buisine N, Tang CM, Chalmers R (2002). Transposon-like Correia elements: Structure, distribution and genetic exchange between pathogenic Neisseria sp. FEBS Lett.

[pgen-0030023-b048] Linz B, Schenker M, Zhu P, Achtman M (2000). Frequent interspecific genetic exchange between commensal *Neisseriae* and Neisseria meningitidis. Mol Microbiol.

[pgen-0030023-b049] Kawai M, Uchiyama I, Kobayashi I (2005). Genome comparison *In Silico* in *Neisseria* suggests integration of filamentous bacteriophages by their own transposase. DNA Res.

[pgen-0030023-b050] Wainwright LA, Frangipane JV, Seifert HS (1997). Analysis of protein binding to the Sma/Cla DNA repeat in pathogenic *Neisseriae*. Nucleic Acids Res.

[pgen-0030023-b051] Aho EL, Urwin R, Batcheller AE, Holmgren AM, Havig K (2005). Neisserial pilin genes display extensive interspecies diversity. FEMS Microbiol Lett.

[pgen-0030023-b052] Aho EL, Botten JW, Hall RJ, Larson MK, Ness JK (1997). Characterization of a class II pilin expression locus from Neisseria meningitidis: Evidence for increased diversity among pilin genes in pathogenic *Neisseria* species. Infect Immun.

[pgen-0030023-b053] Wainwright LA, Pritchard KH, Seifert HS (1994). A conserved DNA sequence is required for efficient gonococcal pilin antigenic variation. Mol Microbiol.

[pgen-0030023-b054] Hodak H, Clantin B, Willery E, Villeret V, Locht C (2006). Secretion signal of the filamentous haemagglutinin, a model two-partner secretion substrate. Mol Microbiol.

[pgen-0030023-b055] Jordan PW, Snyder LA, Saunders NJ (2005). Strain-specific differences in Neisseria gonorrhoeae associated with the phase variable gene repertoire. BMC Microbiol.

[pgen-0030023-b056] Martin P, van de Ven T, Mouchel N, Jeffries AC, Hood DW (2003). Experimentally revised repertoire of putative contingency loci in Neisseria meningitidis strain MC58: Evidence for a novel mechanism of phase variation. Mol Microbiol.

[pgen-0030023-b057] Zhou J, Spratt BG (1992). Sequence diversity within the *argF*, *fbp* and *recA* genes of natural isolates of Neisseria meningitidis: Interspecies recombination within the *argF* gene. Mol Microbiol.

[pgen-0030023-b058] Bonfield JK, Smith K, Staden R (1995). A new DNA sequence assembly program. Nucleic Acids Res.

[pgen-0030023-b059] Berriman M, Rutherford K (2003). Viewing and annotating sequence data with Artemis. Brief Bioinform.

[pgen-0030023-b060] Carver TJ, Rutherford KM, Berriman M, Rajandream MA, Barrell BG (2005). ACT: The Artemis Comparison Tool. Bioinformatics.

[pgen-0030023-b061] Altschul SF, Gish W, Miller W, Myers EW, Lipman DJ (1990). Basic local alignment search tool. J Mol Biol.

[pgen-0030023-b062] Wistrand M, Sonnhammer EL (2005). Improved profile HMM performance by assessment of critical algorithmic features in SAM and HMMER. BMC Bioinformatics.

[pgen-0030023-b063] Saunders NJ, Jeffries AC, Peden JF, Hood DW, Tettelin H (2000). Repeat-associated phase variable genes in the complete genome sequence of Neisseria meningitidis strain MC58. Mol Microbiol.

[pgen-0030023-b064] Jordan P, Snyder LA, Saunders NJ (2003). Diversity in coding tandem repeats in related Neisseria spp. BMC Microbiol.

[pgen-0030023-b065] Hancock JM, Shaw PJ, Bonneton F, Dover GA (1999). High sequence turnover in the regulatory regions of the developmental gene hunchback in insects. Mol Biol Evol.

