# Supplementary material for: Meningococcal Genetic Variation Mechanisms Viewed through Comparative Analysis of Serogroup C Strain FAM18
Source: PLoS Genet. 2007 Feb 16;3(2):e23. doi: 10.1371/journal.pgen.0030023 (PMC1797815; doi:10.1371/journal.pgen.0030023)
Supplement: Supplementary file 1 [file pgen.0030023.sg001.pdf]

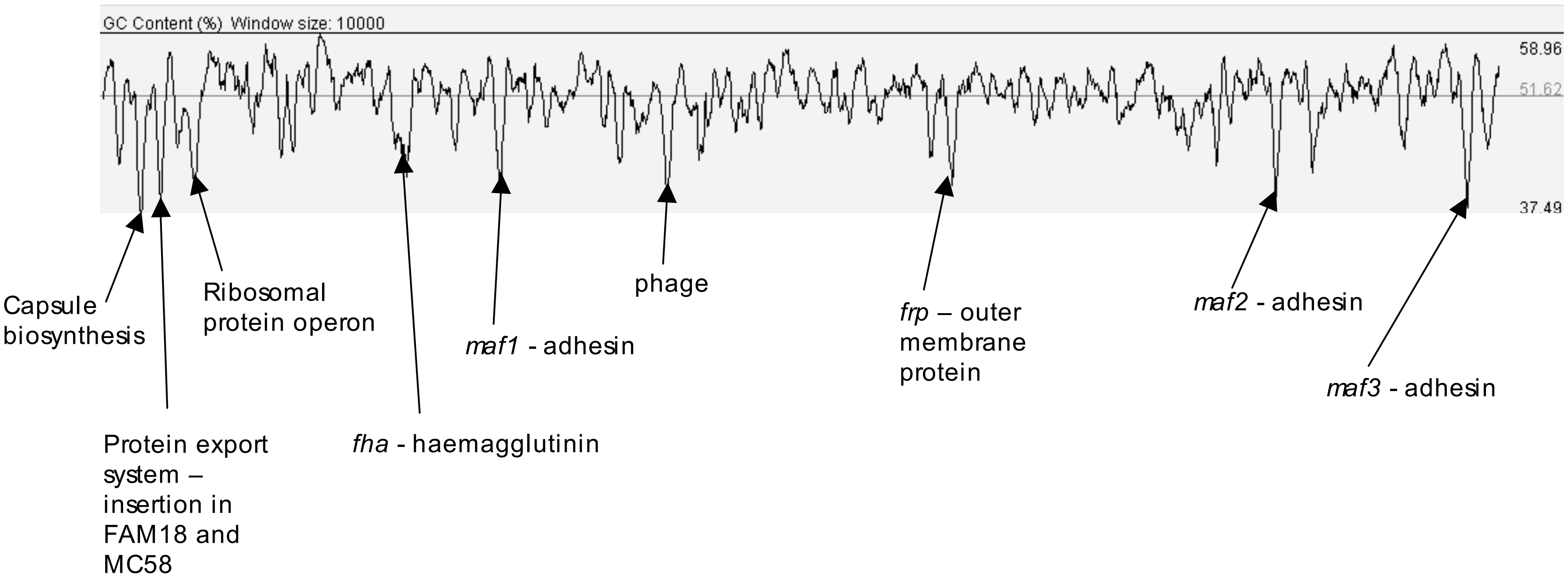

GC Content (%) Window size: 10000
58.96
51.62
37.49
Capsule biosynthesis
Protein export system – insertion in FAM18 and MC58
Ribosomal protein operon
*fha* - haemagglutinin
*maf1* - adhesin
phage
*frp* – outer membrane protein
*maf2* - adhesin
*maf3* - adhesin
