# Supplementary material for: Meningococcal Genetic Variation Mechanisms Viewed through Comparative Analysis of Serogroup C Strain FAM18
Source: PLoS Genet. 2007 Feb 16;3(2):e23. doi: 10.1371/journal.pgen.0030023 (PMC1797815; doi:10.1371/journal.pgen.0030023)
Supplement: Supplementary file 2 [file pgen.0030023.st001.doc]

**Supplementary table 1: Genes unique to each of the three *Neisseria meningitidis* genomes (strains Z2491, MC58 and FAM18) relative to the other two.**

|  | **Locus ID** | **Predicted function** | **Cause of gene complement difference** |
| --- | --- | --- | --- |
| ***N. meningitidis* serogroup B strain MC58** | | |  |
|  | NMB0067 | sialyltransferase SiaD | capsule locus - MME |
|  | NMB0489 | hypothetical protein | integration |
|  | NMB0490 | PspA-related protein | integration |
|  | NMB0491 | hypothetical protein | integration |
|  | NMB0492 | hypothetical protein | integration |
|  | NMB0493 | Hemagglutinin/hemolysin-related protein | integration |
|  | NMB0495 | replication protein | integration |
|  | NMB0499 | hypothetical protein | integration |
|  | NMB0500 | hypothetical protein | integration |
|  | NMB0501 | hypothetical protein | integration |
|  | NMB0511 | hypothetical protein | integration |
|  | NMB0512 | hypothetical protein | integration |
|  | NMB0513 | hypothetical protein | integration |
|  | NMB0725 | modification methylase HgaI-1 | MME |
|  | NMB0726 | type II restriction enzyme HgaI | MME |
|  | NMB0727 | N-6 adenine-specific DNA methylase | MME |
|  | NMB0780 | hypothetical protein | MME |
|  | NMB0831 | Restriction specificity protein | MME |
|  | NMB0832 | anticodon nuclease | MME |
|  | NMB0961 | funZ protein | MME |
|  | NMB1005 | hypothetical protein | IS150-associated region |
|  | NMB1006 | hypothetical protein | IS150-associated region |
|  | NMB1007 | transcriptional regulator | IS150-associated region |
|  | NMB1008 | hypothetical protein | IS150-associated region |
|  | NMB1009 | conserved hypothetical protein | IS150-associated region |
|  | NMB1010 | hypothetical protein | IS150-associated region |
|  | NMB1011 | hypothetical protein | IS150-associated region |
|  | NMB1084 | hypothetical protein | phage transposase-associated gene |
|  | NMB1093 | hypothetical protein | MME |
|  | NMB1100 | hypothetical protein | IS30-associated phage region |
|  | NMB1101 | conserved hypothetical protein | IS30-associated phage region |
|  | NMB1102 | hypothetical protein | IS30-associated phage region |
|  | NMB1103 | hypothetical protein | IS30-associated phage region |
|  | NMB1104 | phage sheath protein | IS30-associated phage region |
|  | NMB1105 | hypothetical protein | IS30-associated phage region |
|  | NMB1106 | hypothetical protein | IS30-associated phage region |
|  | NMB1107 | hypothetical protein | IS30-associated phage region |
|  | NMB1108 | hypothetical protein | IS30-associated phage region |
|  | NMB1208 | Doubtful CDS | MME |
|  | NMB1209 | Doubtful CDS | MME |
|  | NMB1210 | toxin-activating protein, putative | MME |
|  | NMB1211 | hypothetical protein | MME |
|  | NMB1212 | hypothetical protein | MME |
|  | NMB1213 | hypothetical protein | MME |
|  | NMB1214 | hemagglutinin/hemolysin-related protein | MME |
|  | NMB1215 | hypothetical protein | MME |
|  | NMB1264 | conserved hypothetical protein | MME |
|  | NMB1265 | conserved hypothetical protein | MME |
|  | NMB1300 | cytidylate kinase | MME |
|  | NMB1311 | hypothetical protein | MME |
|  | NMB1329 | hypothetical protein | MME |
|  | NMB1343 | hypothetical protein | MME |
|  | NMB1408 | hypothetical protein | IS1016-associated region |
|  | NMB1409 | FrpA/C-related protein | IS1016-associated region |
|  | NMB1410 | hypothetical protein | IS1016-associated region |
|  | NMB1495 | Doubtful CDS | IS1106-associated region |
|  | NMB1496 | conserved hypothetical protein | IS1106-associated region |
|  | NMB1502 | hypothetical protein | MME |
|  | NMB1596 | hypothetical protein | IS1106-associated region |
|  | NMB1597 | hypothetical protein | IS1106-associated region |
|  | NMB1598 | hypothetical protein | IS1106-associated region |
|  | NMB1599 | hypothetical protein | IS1106-associated region |
|  | NMB1692 | chorismate mutase-related protein | MME |
|  | NMB1696 | acyl carrier protein | deletion / degradation |
|  | NMB1697 | acyl carrier protein, putative | deletion / degradation |
|  | NMB1698 | acyltransferase, putative | deletion / degradation |
|  | NMB1700 | hypothetical protein | deletion / degradation |
|  | NMB1701 | hypothetical protein | deletion / degradation |
|  | NMB1702 | 3-oxoacyl-(acyl-carrier-protein) reductase | deletion / degradation |
|  | NMB1753 | VapD-related protein | piv-associated region |
|  | NMB1754 | cryptic plasmid protein A-related protein | piv-associated region |
|  | NMB1755 | hypothetical protein | piv-associated region |
|  | NMB1756 | hypothetical protein | piv-associated region |
|  | NMB1757 | hypothetical protein | piv-associated region |
|  | NMB1758 | hypothetical protein | piv-associated region |
|  | NMB1759 | conserved hypothetical protein | piv-associated region |
|  | NMB1760 | conserved hypothetical protein | piv-associated region |
|  | NMB1761 | conserved hypothetical protein | piv-associated region |
|  | NMB1763 | toxin-activating protein, putative | IS30-associated region |
|  | NMB1764 | hypothetical protein | IS30-associated region |
|  | NMB1765 | hypothetical protein | IS30-associated region |
|  | NMB1766 | hypothetical protein | IS30-associated region |
|  | NMB1767 | hypothetical protein | IS30-associated region |
|  | NMB1768 | hemagglutinin/hemolysin-related protein | IS30-associated region |
|  | NMB1771 | hypothetical protein | IS30-associated region |
|  | NMB1772 | hypothetical protein | IS30-associated region |
|  | NMB1773 | hypothetical protein | IS30-associated region |
|  | NMB1774 | hypothetical protein | IS30-associated region |
|  | NMB1775 | hypothetical protein | IS30-associated region |
|  | NMB1848 | Doubtful CDS | integration |
|  | NMB1850 | hypothetical protein | deletion / degradation |
|  | NMB1854 | hypothetical protein | deletion / degradation |
|  | NMB2008 | chloride channel protein-related protein | integration |
|  | NMB2012 | transcriptional regulator, HTH_3 family | *comP*-associated region |
|  | NMB2013 | hypothetical protein | *comP*-associated region |
| ***N. meningitidis* serogroup A strain Z2491** | | |  |
|  | NMA0001 | hypothetical protein NMA0001 | MME |
|  | NMA0095 | hypothetical protein NMA0095 | MME |
|  | NMA0096 | hypothetical protein NMA0096 | MME |
|  | NMA0131 | hypothetical protein NMA0131 | MME |
|  | NMA0132 | hypothetical protein NMA0132 | MME |
|  | NMA0132A | hypothetical protein NMA0132A | MME |
|  | NMA0179 | hypothetical protein NMA0180 | integration |
|  | NMA0199 | putative UDP-N-acetyl-D-glucosamine 2-epimerase | capsule - MME |
|  | NMA0200 | putative capsule biosynthesis protein | capsule - MME |
|  | NMA0201 | putative capsule biosynthesis protein | capsule - MME |
|  | NMA0202 | putative capsule biosynthesis protein | capsule - MME |
|  | NMA0629 | hypothetical protein NMA0629 | IS1106-associated region |
|  | NMA0630 | hypothetical protein NMA0630 | IS1106-associated region |
|  | NMA0631 | hypothetical protein NMA0631 | IS1106-associated region |
|  | NMA0935 | hypothetical protein NMA0935 | MME |
|  | NMA0936 | hypothetical protein NMA0936 | MME |
|  | NMA1040 | putative DNA-binding protein | MME |
|  | NMA1155 | hypothetical protein NMA1155 | MME |
|  | NMA1156 | hypothetical protein NMA1156 | MME |
|  | NMA1157 | hypothetical protein NMA1157 | MME |
|  | NMA1158 | hypothetical protein NMA1158 | MME |
|  | NMA1210 | hypothetical protein NMA1210 | IS1106-associated region |
|  | NMA1211 | hypothetical protein NMA1211 | IS1106-associated region |
|  | NMA1215 | hypothetical protein NMA1215 | IS1106-associated region |
|  | NMA1292 | hypothetical protein NMA1292 | phage transposase-associated gene |
|  | NMA1293 | putative phage anti-repressor protein | phage transposase-associated gene |
|  | NMA1294 | hypothetical protein NMA1294 | phage transposase-associated gene |
|  | NMA1295 | hypothetical protein NMA1295 | phage transposase-associated gene |
|  | NMA1296 | hypothetical protein NMA1296 | phage transposase-associated gene |
|  | NMA1297 | hypothetical protein NMA1297 | phage transposase-associated gene |
|  | NMA1298 | hypothetical protein NMA1298 | phage transposase-associated gene |
|  | NMA1299 | hypothetical protein NMA1299 | phage transposase-associated gene |
|  | NMA1300 | putative membrane protein | phage transposase-associated gene |
|  | NMA1544 | hypothetical protein | MME |
|  | NMA1587 | hypothetical protein | MME |
|  | NMA1789 | hypothetical protein | integration |
|  | NMA1791 | hypothetical protein | integration |
|  | NMA1821 | conserved hypothetical protein | integration |
|  | NMA1822 | hypothetical protein | integration |
|  | NMA1823 | hypothetical protein | integration |
|  | NMA1824 | hypothetical protein | integration |
|  | NMA1825 | hypothetical protein | integration |
|  | NMA1826 | hypothetical protein | integration |
|  | NMA1827 | hypothetical protein | integration |
|  | NMA1828 | hypothetical protein | integration |
|  | NMA1829 | hypothetical protein | integration |
|  | NMA1830 | hypothetical protein | integration |
|  | NMA1831 | hypothetical protein | integration |
|  | NMA1832 | hypothetical protein | integration |
|  | NMA1833 | hypothetical DNA-binding protein | integration |
|  | NMA1835 | hypothetical protein | integration |
|  | NMA1836 | hypothetical protein | integration |
|  | NMA1837 | hypothetical protein | integration |
|  | NMA1838 | hypothetical DNA-binding protein | integration |
|  | NMA1840 | hypothetical protein | integration |
|  | NMA1841 | hypothetical protein | integration |
|  | NMA1842 | hypothetical protein | integration |
|  | NMA1843 | hypothetical protein | integration |
|  | NMA1844 | hypothetical protein | integration |
|  | NMA1845 | hypothetical protein | integration |
|  | NMA1847 | conserved hypothetical protein | integration |
|  | NMA1848 | hypothetical protein | integration |
|  | NMA1849 | hypothetical protein | integration |
|  | NMA1850 | hypothetical protein | integration |
|  | NMA1853 | hypothetical protein | MME |
|  | NMA1865 | hypothetical protein | phage-associated region |
|  | NMA1869 | hypothetical protein | phage-associated region |
|  | NMA1870 | hypothetical protein | phage-associated region |
|  | NMA1871 | hypothetical protein | phage-associated region |
|  | NMA1873 | hypothetical protein | phage-associated region |
|  | NMA1874 | hypothetical protein | phage-associated region |
|  | NMA1875 | hypothetical protein | phage-associated region |
|  | NMA1876 | hypothetical protein | phage-associated region |
|  | NMA1877 | putative small secreted protein | phage-associated region |
|  | NMA1878 | hypothetical protein | phage-associated region |
|  | NMA1879 | hypothetical protein | phage-associated region |
|  | NMA1880 | hypothetical protein | phage-associated region |
|  | NMA1881 | hypothetical DNA-binding protein | phage-associated region |
|  | NMA1882 | putative transposase | phage-associated region |
|  | NMA1883 | Ner-like DNA-binding protein | phage-associated region |
|  | NMA2095 | putative integral membrane efflux protein | integration |
|  | NMA2096 | putative membrane pump-driving ATPase | integration |
|  | NMA2230 | hypothetical protein | MME |
| ***N. meningitidis* serogroup C strain FAM18** | | |  |
|  | NMC0050 | conserved hypothetical protein | capsule - MME |
|  | NMC0051 | alpha-2,8-polysialyltransferase | capsule - MME |
|  | NMC0208 | Doubtful CDS | MME |
|  | NMC0209 | conserved hypothetical protein | MME |
|  | NMC0210 | class II pilin PilE | MME |
|  | NMC0295 | putative DNA methylase | IS1106-associated region |
|  | NMC0296 | conserved hypothetical protein (pseudogene) | IS1106-associated region |
|  | NMC0298 | hypothetical protein | IS1106-associated region |
|  | NMC0327 | putative DNA adenine methylase | Native gene *dam* - the *drg* (*dam* replacement gene) is present in MC58 and Z2491 |
|  | NMC0328 | putative Modification methylase DpnIIB | *dam*-associated gene |
|  | NMC0329 | hypothetical protein | *dam*-associated gene |
|  | NMC0393 | hypothetical protein | MME |
|  | NMC0398 | pilin glycosylation protein | MME |
|  | NMC0399 | Pglb2 | MME |
|  | NMC0678 | putative 50S ribosomal protein L20 | MME |
|  | NMC0679 | putative modification methylase NmeDI | MME |
|  | NMC0680 | Putative type II restriction enzyme NmeDIP | MME |
|  | NMC0852 | hypothetical protein | lambda 'phage |
|  | NMC0853 | hypothetical protein | lambda 'phage |
|  | NMC0854 | hypothetical protein | lambda 'phage |
|  | NMC0855 | hypothetical protein | lambda 'phage |
|  | NMC0856 | hypothetical protein | lambda 'phage |
|  | NMC0857 | putative phage related protein | lambda 'phage |
|  | NMC0858 | putative phage-related protein | lambda 'phage |
|  | NMC0860 | hypothetical protein | lambda 'phage |
|  | NMC0861 | hypothetical protein | lambda 'phage |
|  | NMC0862 | hypothetical protein | lambda 'phage |
|  | NMC0863 | hypothetical protein | lambda 'phage |
|  | NMC0865 | putative prophage-related protein | lambda 'phage |
|  | NMC0866 | putative phage-related protein | lambda 'phage |
|  | NMC0867 | putative phage-related protein | lambda 'phage |
|  | NMC0868 | putative phage-related protein NLPC_P60 | lambda 'phage |
|  | NMC0869 | putative phage-related protein | lambda 'phage |
|  | NMC0870 | Neisseria meningitidis phage 2120.; ORF 4 | lambda 'phage |
|  | NMC0871 | hypothetical protein | lambda 'phage |
|  | NMC0872 | putative integral membrane protein | lambda 'phage |
|  | NMC0873 | hypothetical protein | lambda 'phage |
|  | NMC0874 | putative membrane protein | lambda 'phage |
|  | NMC0875 | putative membrane protein | lambda 'phage |
|  | NMC0877 | putative ATP binding protein | lambda 'phage |
|  | NMC0885 | hypothetical protein | IS1106-associated region |
|  | NMC0887 | putative HlyD family secretion protein | IS1106-associated region |
|  | NMC0889 | putative integral membrane protein | IS1106-associated region |
|  | NMC0890 | putative integral membrane protein | IS1106-associated region |
|  | NMC0893 | putative ParB protein | IS1106-associated region |
|  | NMC0939 | hypothetical protein | IS1106-associated region |
|  | NMC0940 | putative ATP-dependent helicase | MME |
|  | NMC0985 | putative oxidoreductase | MME |
|  | NMC1053 | hypothetical protein | integration |
|  | NMC1117 | hypothetical protein | MME |
|  | NMC1118 | putative transcriptional regulator | MME |
|  | NMC1225 | hypothetical protein | MME |
|  | NMC1268 | hypothetical protein | MME |
|  | NMC1519 | hypothetical protein | IS1106-associated region |
|  | NMC1520 | C-5 cytosine-specific DNA methylase | IS1106-associated region |
|  | NMC1626 | hypothetical protein (fragment) | MME |
|  | NMC1627 | putative acetyltransferase (pseudogene) | MME |
|  | NMC1730 | putative integral membrane protein | MME |
|  | NMC1731 | putative D-alanyl-d-alanine dipeptidase | MME |
