# Supplementary material for: Meningococcal Genetic Variation Mechanisms Viewed through Comparative Analysis of Serogroup C Strain FAM18
Source: PLoS Genet. 2007 Feb 16;3(2):e23. doi: 10.1371/journal.pgen.0030023 (PMC1797815; doi:10.1371/journal.pgen.0030023)
Supplement: Supplementary file 3 [file pgen.0030023.st002.doc]

**Supplementary Table 2: Repeat arrays and functional annotation of flanking genes in FAM18.** Flanking gene cells are coloured according to functional classification (see key below).

| **Genomic position** | **Array size (bp)** | **Repeats types present** | **Flanking Gene (left)** | **Flanking gene (right)** |
| --- | --- | --- | --- | --- |
| 973809..976403 | 2595 | 19 NIME, 2 CORREIA | NMC0978; putative surface fibril protein | NMC0980; putative rubredoxin |
| 1486854..1489385 | 2532 | 29 NIME, 2 CORREIA, 1 REP4 | NMC1469, lbpB; lactoferrin binding protein | NMC1470; Remnant of transposase from insertion element IS1106A |
| 1927980..1929962 | 1983 | 18 NIME | NMC1899; conserved hypothetical protein | NMC1900, lgtB2; lacto-N-neotetraose biosynthesis glycosyl tranferase |
| 1374978..1376888 | 1911 | 19 NIME | NMC1380; conserved hypothetical protein | NMC1381; Putative transposase for IS1655 |
| 2070654..2072474 | 1821 | 19 NIME, 1 CORREIA | NMC2020, porB; porin, major outer membrane protein P.I | NMC2021, thiC; thiamin biosynthesis protein |
| 1998924..2000735 | 1812 | 8 NIME, 4 CORREIA, 1 REP5 | NMC1962, thdF; probable thiophene and furan oxidation protein | NMC1963, feta; putative iron-regulated outer membrane protein |
| 841731..843416 | 1686 | 17 NIME, 2 CORREIA | NMC0831; putative pilin | NMC0832; conserved hypothetical protein |
| 1752462..1754012 | 1551 | 19 NIME | NMC1719; Opa1800 outer membrane protein precursor | NMC1720; conserved hypothetical protein |
| 375180..376685 | 1500 | 8 NIME, 1 REP4 | NMC0370, carA; carbamoyl phosphate synthase small subunit | NMC0371, pilC1; pilus-associated protein |
| 1726185..1727600 | 1416 | 16 NIME | NMC1691, tbpB; transferrin-binding protein B | NMC1692, glr; glutamate racemase |
| 1480317..1481660 | 1344 | 18 NIME | NMC1467; putative transposase for IS1106A3 | NMC1468, lbpA; lactoferrin binding protein A |
| 522024..523331 | 1308 | 10 NIME, 2 CORREIA, 1 REP4, 1REP5 | NMC0494; putative transposase | NMC0495, dnak; putative chaperone protein |
| 1741977..1743230 | 1254 | 13 NIME, 1 CORREIA, 1 REP5 | NMC1708; putative integral membrane protein | NMC1709; hypothetical protein |
| 945762..947006 | 1245 | 13 NIME, 1 CORREIA | NMC0942; putative membrane protein | NMC0944; putative outer-membrane receptor protein |
| 1565043..1566242 | 1200 | 13 NIME | NMC1544, rnhA; ribonuclease HI | NMC1545; hypothetical protein |
| 423408..424479 | 1172 | 10 NIME, 1 CORREIA, 1 REP5 | NMC0405; conserved hypothetical protein | NMC0406, aroB; 3-dehydroquinate synthase |
| 1430553..1431716 | 1164 | 8 NIME, 2 CORREIA, 1 REP3, 1 REP4 | NMC1426; putative integral membrane protein (CstA-like) | NMC1428; putative outer membrane substrate binding protein |
| 1550733..1551887 | 1155 | 11 NIME | NMC1528; conserved hypothetical protein | NMC1529; hypothetical protein |
| 278799..279950 | 1152 | 14 NIME | NMC0276; putative rotamase | NMC0277; hypothetical protein |
| 1434819..1435955 | 1137 | 13 NIME, 1 CORREIA | NMC1428; putative outer membrane substrate binding protein | NMC1429, lysC; aspartate kinase |
| 396768..397868 | 1101 | 10 NIME, 1 ATR, 1 CORREIA | NMC0383, ileS; putative isoleucyl-tRNA synthetase | NMC0384, lsp; putative lipoprotein signal peptidase |
| 588975..590075 | 1100 | 1 REP2, 3 NIME, 1 CORREIA, 1 REP3, 1 ATR | NMC0557, amtB; putative ammonium transporter | NMC0558; putative IS1106 transposase (pseudogene) |
| 892611..893702 | 1092 | 8 NIME, 2 CORREIA, 1 REP5, 1 REP4 | NMC0896; Putative transposase for IS1106A3 (fragment) | NMC0897, icd; putative isocitrate dehydrogenase |
| 1402239..1403330 | 1092 | 14 NIME | NMC1402; conserved hypothetical protein | NMC1403, opaA; pseudogene (opacity protein) |
| 899832..900878 | 1047 | 13 NIME | NMC0902; putative acyl-CoA hydrolase | NMC0903, opaD; pseudogene (opacity protein) |
| 1573617..1574654 | 1038 | 9 NIME, 2 CORREIA, 1 REP3, 1 REP4 | NMC1550; hypothetical protein | NMC1551, opaB; pseudogene (opacity protein) |
| 1283607..1284635 | 1029 | 13 NIME | NMC1297; putative integral membrane transporter | NMC1298, xseA; exonuclease VII large subunit |
| 1990191..1991201 | 1011 | 3 NIME, 2 CORREIA, 1 REP3, 1 ATR | NMC1957, polA; DNA polymerase I | NMC1958; conserved hypothetical protein |
| 2076990..2077991 | 1002 | 11 NIME | NMC2023; ABC-transport system ATP-binding protein | NMC2024, ptsI; phosphoenolpyruvate-protein phosphotransferase |
| 454602..455585 | 984 | 5 NIME, 1 CORREIA | NMC0433, recG; putative DNA helicase | NMC0434, argC; putative N-acetyl-gamma-glutamyl-phosphate reductase |

**Colour codes:**

**white:** Pathogenicity/adaptation

**grey:** Energy metabolism

**red:** Information transfer

**dark green:** Surface/exported

**sky blue:** Degradation of large molecules

**yellow:** Central metabolism

**light green:** Unknown

**orange:** Conserved hypothetical

**pink:** Phage/IS elements
