# Supplementary material for: Meningococcal Genetic Variation Mechanisms Viewed through Comparative Analysis of Serogroup C Strain FAM18
Source: PLoS Genet. 2007 Feb 16;3(2):e23. doi: 10.1371/journal.pgen.0030023 (PMC1797815; doi:10.1371/journal.pgen.0030023)
Supplement: Supplementary file 4 [file pgen.0030023.st003.doc]

**Supplementary Table 3: Phase variable genes of the *Neisseria* spp.**

| **Candidacy *** | **gene** | **annotated function** | **NMC# †** | **XNG# †** | **NMB# †** | **NMA# †** | **Repeat tract ‡** | **Reference(s)** |
| --- | --- | --- | --- | --- | --- | --- | --- | --- |
| K | *opa* | opacity protein | 4 copies of the gene | 11 copies of the gene | 4 copies of the gene | 3 copies of the gene | (CTTCT) c | ^1^ |
| K | *opc* | class 5 outer membrane protein | -------------- | -------------- | NMB1053 | NMA1251 | (C) p | ^2^ |
| K | *porA* | outer membrane protein | NMC1364 | XNG0832 | NMB1429 | NMA1642 | (T)C(G) or (T)GC(G) p | ^3^ |
| K | *nadA* | putative adhesin / invasin | NMC1969 | -------------- | NMB1994 | -------------- | (TAAA) p | ^4^ |
| K | *pilC* | pilin biogenesis protein | NMC0033 & NMC0371 | XNG0049 & XNG1797 | NMB0049 & NMB1847 | NMA0293 & NMA0609 | (G) & (G) c | ^5^ |
| K | *pgtA* / *pglA* | pilin glycosylation protein | NMC0213 | XNG1644 | NMB0218 | NMA0048 | (G) c | ^6^ |
| K | *pglE* | pilin glycosylation protein | NMC0568 | XNG0189 | NMB0624 | NMA0832 | (CAAACAA) or (CAAACAC) c | ^7^ |
| K | *pglG* | pilin glycosylation protein | NMC0401 | XNG0081 | -------------- | NMA0641 | (C) c | ^8^ |
| K | *pglH* | pilin glycosylation protein | NMC0400 | XNG0080 | NMB1819 | NMA0640 | (C) c | ^8^ |
| K | *dca*/*pptA* | competence-associated protein / pilin phosphorylcholine transferase | NMC1750 | XNG1423 | NMB0415 | NMA2069 | (G) c | ^9^, ^10^ |
| K | *lgtA* | acto-N-neotetraose biosynthesis glycosyl transferase | NMC1902 | XNG2045 | NMB1929 | NMA0524 | (G) c | ^11, 12^ |
| K | *lgtC* | lipopolysaccharide biosynthesis protein | -------------- | XNG2047 | -------------- | -------------- | (G) c | ^11, 12^ |
| K | *lgtD* | lipopolysaccharide biosynthesis protein | -------------- | XNG2048 | -------------- | -------------- | (G) c | ^11^ ^12^ |
| K | *lgtG* | lipopolysaccharide glycosyl transferase | NMC2011 | XNG1968 | NMB2032 | -------------- | (C) c | ^13^ |
| K | *siaD* | capsule biosynthesis protein | -------------- | -------------- | NMB0067 | -------------- | (C) c | ^14^ |
| K | *hpuA* | hemoglobin-haptoglobin utilization protein | NMC1946 | XNG2004 | -------------- | NMA0475 | (G) c | ^15^ |
| K | *hmbR* | hemoglobin receptor | NMC1586 | XNG1216 | NMB1668 | NMA1925 | (G) c | ^16^ |
| K | *frpB / fetA* | iron-regulated outer membrane protein | NMC1963 | XNG1989 | NMB1988 | NMA0453 | (C) p | ^17^ |
| K |  | Type III restriction system methylase | NMC1194 | XNG0520 | NMB1261 | NMA1467 | (CCCAA) c | ^8^ |
| K |  | putative restriction system methylase | NMC1310 | XNG0612 | NMB1375 | NMA1590 | (GCCA) c | ^8^ |
| K |  | hypothetical protein | -------------- | XNG0503a | -------------- | -------------- | (C)A(C) c | ^8^ |
| K |  | hypothetical protein § | -------------- | XNG1733 | -------------- | -------------- | (G) c | ^8^ |
| K |  | hypothetical protein \|\| | unannotated 3' of NMC0040 | XNG1788 | NMB0297 | NMA0212 | (G) c | ^8^ |
| K |  | hypothetical protein | unannotated between NMC2114 and NMC2116 | XNG1834 | -------------- | NMA0223 | (C) c | ^8^ |
| S | *virG* | AIDA related protein | NMC1454 | XNG0905 | NMB1525 | NMA1725 | (AAGC) c |  |
| S | *vapA* | virulence associated protein | NMC1859 | XNG1563 | NMB0312 | NMA2175 | (AAGC) c |  |
| S |  | adhesin | -------------- | XNG1341 | -------------- | -------------- | (CAAG) c |  |
| S | *pglB*2 | pilin glycosylation protein | NMC0399 | -------------- | -------------- | -------------- | (A) c |  |
| S |  | putative acetyltransferase | NMC2013 | -------------- | -------------- | NMA0407 | (G) c |  |
| S | *wbpC / pglI* | putative lipopolysaccharide biosynthesis or pilin glycosylation protein | NMC0380 | XNG0060 | NMB1836 | NMA0619 | (G) c |  |
| S |  | putative glycosyl transferase | NMC1156 | XNG1041** | NMB1255 | NMA1427 | (G) c |  |
| S | *lbpA* | lactoferrin-binding protein A | NMC1468 | XNG0245 | NMB1540 | NMA1739 | (G) c |  |
| S | *hsdS* | type I restriction enzyme S protein | -------------- | XNG0388 | NMB0831 | NMA1040 | (G) c |  |
| S | *aspA* | putative serotype-1-specific antigen | NMC1943 | XNG0986 | NMB1969 | NMA0478 | (C) c |  |
| S |  | putative lipoprotein | NMC0009 | XNG1934 | NMB0032 | NMA0277 | (A) p |  |
| S | *mtfB* | ABC transporter ATP-binding component | NMC0086 | -------------- | NMB0098 | -------------- | (G) c |  |
| S |  | putative hydrolase | NMC0016 | XNG1942/XNG1941 | NMB0040/NMB0039 | NMA0286/NMA0285 | (C) c |  |
| S |  | hypothetical protein | unannotated between NMC1436 and NMC1437 | XNG0887 | NMB1507 | unannotated between NMA1707 and NMA1708 (NMA1707a) | (AAGC) c |  |
| S |  | hypothetical protein | NMC1801 | XNG1281 | NMB0368 | NMA2117 | (A) c |  |
| S |  | hypothetical protein | NMC0436 | XNG0112 | NMB1786 | NMA0679/NMA0678 | (A) p |  |
| S |  | hypothetical protein | NMC0804 | XNG0412 | -------------- | NMA1077 | (G) c |  |
| S |  | hypothetical protein | NMC0049 | -------------- | NMB0065 | -------------- | (T) c |  |
| S |  | hypothetical protein †† | -------------- | XNG0470 | -------------- | -------------- | (G) c |  |
| S |  | hypothetical protein | -------------- | XNG0473 | -------------- | -------------- | (T) c |  |
| S |  | hypothetical protein | -------------- | XNG1000 | -------------- | -------------- | (G) c |  |
| S |  | hypothetical protein \|\| | -------------- | XNG1207a | -------------- | -------------- | (G) c |  |
| S |  | hypothetical protein | -------------- | XNG1511 | -------------- | -------------- | (G) c |  |
| S |  | hypothetical protein \|\| | -------------- | XNG1577 | -------------- | -------------- | (G) c |  |
| S |  | hypothetical protein | -------------- | -------------- | NMB1893 | NMA0562 | (TTCC) c | ^18^ |
| S ‡‡ |  | hypothetical protein | -------------- | -------------- | -------------- | NMA0132 | (C) p |  |
| M | *nifS* | aminotransferase | NMC1315 | XNG0608 | NMB1379 | NMA1594 | (C) c |  |
| M | *pntA* | NAD(P) transhydrogenase alpha subunit | NMC0960 | XNG1356 | NMB0980 | NMA1177 | (C) p |  |
| L |  | modification methylase NlaIV | NMC1180 | XNG0644 | NMB1033 | NMA1453 | (A) c |  |
| L | *rplK* | 50S ribosomal protein L11 | NMC0119 | XNG1740 | NMB0127 | NMA0146 | (C) c |  |
| L | *dinG* | probable ATP-dependent helicase | NMC0293 | XNG1589 | NMB0287 | NMA2200 | (C) c |  |
| L | *plsX* | fatty acid-phospholipid synthesis protein | NMC0310 | XNG2061 | NMB1913 | NMA0542 | (TTCC) c |  |
| L |  | prolyl oligopeptidase family protein | NMC0342 | XNG0023 | NMB1877 | NMA0579 | (G) c |  |
| L | *potD*-2 | spermidine/putrescine ABC transporter, periplasmic component | NMC0567 | XNG0188 | NMB0623 | NMA0831 | (C) c |  |
| L | *lldD* | L-lactate dehydrogenase | NMC1312 | XNG0610 | NMB1377 | NMA1592 | (G) c |  |
| L | *frpC* | iron regulated protein FrpC | NMC1346 | -------------- | NMB1407 | -------------- | (ATAACAAA) c |  |
| L | *dnaX* | DNA polymerase III, subunits gamma and tau | NMC1379 | XNG0703 | NMB1443 | NMA1656 | (C) c |  |
| L | *ppx* | exopolyphosphatase | NMC1405 | XNG0954 | NMB1467 | NMA1679 | (C) c |  |
| L | *amiC* | N-acetylmuramoyl-L-alanine amidase | NMC1694 | XNG1384 | NMB0456 | NMA2028 | (C) c |  |
| L §§ | *ner* | regulator | -------------- | -------------- | NMB1080 | -------------- | (A) c |  |
| L |  | ABC transporter | -------------- | -------------- | NMB2008 | -------------- | (C) p |  |
| L |  | hypothetical protein | NMC0394 | XNG0075 | NMB1824 | NMA0635 | (C) c |  |
| L |  | hypothetical protein | NMC0535 | XNG0158 | NMB0593 | NMA0796 | (C) c |  |
| L |  | hypothetical protein | NMC1161 | XNG0663 | -------------- | NMA1436 | (C) & (T) c |  |
| L \|\|\|\| |  | hypothetical protein | NMC1205 | XNG0508 | NMB1275 | NMA1480 | (AGCA) c |  |
| L |  | hypothetical protein | NMC1265 | XNG0552 | NMB1327 | NMA1541 | (C) c |  |
| L |  | hypothetical protein *** | NMC1424 | XNG0973 | NMB0488 | -------------- | (C) c |  |
| L |  | hypothetical protein | NMC1788 | XNG1460 | NMB0377 | NMA2111 | (C) c |  |
| L |  | hypothetical protein *** | -------------- | XNG0973 | NMB1489 | -------------- | (C) c |  |
| L |  | hypothetical protein | -------------- | -------------- | NMB1760 | -------------- | (C) c |  |

* The predicted likelihood of phase variability. K indicates a known phase variable gene; S indicates a strong candidate for phase variation, which can be based on repeat length, evidence of variation in repeat length between strains, and similarity to repeats known to be phase variable; M indicates a moderate candidate for which there is less supporting evidence than for strong candidates; L indicates a low candidate, which includes genes that contain a relatively short repeat, particularly if no variation in the repeat has been shown between strains or if the length and nature of the repeat suggest that it cannot readily reversibly change.

† CDS designation numbers come from the appropriate annotations. NMC#: *N. meningitidis* strain FAM18 (this publication); XNG#: *N. gonorrhoeae* strain FA1090 (annotation available from https://gbrowse.molbiol.ox.ac.uk/cgi-bin/gbrowse/FA1090); NMB#: *N. meningitidis* strain MC58 (Tettelin *et al*., 2000); NMA#: *N. meningitidis* strain Z2491 (Parkhill *et al*., 2000). When no CDS number is listed, no homologue is present.

‡ The simple sequence repeat which makes up the known or potential phase variable repeat tract. The repeated base or bases are in parentheses. When more than one repeat tract is present in the gene, these are designated with ‘&’. When different bases make up the repeat when it is present, the alternative repeats are shown, these are designated with ‘or’. Following the sequence a ‘c’ indicates that the repeat is in the coding region and a ‘p’ indicates that the repeat is in the promoter, or predicted promoter, region.

§ This CDS has weak homology to a small portion of NMA0586 and NMB1870.

|| XNG1207a, XNG1577, and XNG1788 are homologues of each other.

** There has been an insertion of *piv* into this CDS, which has also been observed in *N. gonorrhoeae* strains FA19, F62, and MS11 (Jordan P, *et al*., *BMC Microbiol*, 2005).

†† XNG0470 is a homologue of XNG1014, and XNG1513 which have a (G)6 and are not frame-shifted.

‡‡ Candidacy interpretation depends upon the location of the promoter.

§§ Candidacy interpretation depends on the initiation codon selection.

|||| This tetrameric motif is known to be expanded and unstable in other contexts in *Neisseria* and *Haemophilus*, but on the standard selection criteria alone would not be considered phase variable.

*** These CDSs are homologues of each other.

1.	Stern, A., Brown, M., Nickel, P. & Meyer, T. F. Opacity genes in Neisseria gonorrhoeae: control of phase and antigenic variation. Cell 47, 61-71 (1986).

2.	Sarkari, J., Pandit, N., Moxon, E. R. & Achtman, M. Variable expression of the Opc outer membrane protein in Neisseria meningitidis is caused by size variation of a promoter containing poly-cytidine. Mol Microbiol 13, 207-17 (1994).

3.	van der Ende, A. et al. Variable expression of class 1 outer membrane protein in Neisseria meningitidis is caused by variation in the spacing between the -10 and -35 regions of the promoter. J Bacteriol 177, 2475-80 (1995).

4.	Martin, P., Makepeace, K., Hill, S. A., Hood, D. W. & Moxon, E. R. Microsatellite instability regulates transcription factor binding and gene expression. Proc Natl Acad Sci U S A 102, 3800-4 (2005).

5.	Jonsson, A. B., Nyberg, G. & Normark, S. Phase variation of gonococcal pili by frameshift mutation in pilC, a novel gene for pilus assembly. Embo J 10, 477-88 (1991).

6.	Banerjee, A. et al. Implications of phase variation of a gene (pgtA) encoding a pilin galactosyl transferase in gonococcal pathogenesis. J Exp Med 196, 147-62 (2002).

7.	Power, P. M. et al. Genetic characterization of pilin glycosylation and phase variation in Neisseria meningitidis. Mol Microbiol 49, 833-47 (2003).

8.	Jordan, P. W., Snyder, L. A. & Saunders, N. J. Strain-specific differences in Neisseria gonorrhoeae associated with the phase variable gene repertoire. BMC Microbiol 5, 21 (2005).

9.	Snyder, L. A., Saunders, N. J. & Shafer, W. M. A putatively phase variable gene (dca) required for natural competence in Neisseria gonorrhoeae but not Neisseria meningitidis is located within the division cell wall (dcw) gene cluster. J Bacteriol 183, 1233-41 (2001).

10.	Warren, M. J. & Jennings, M. P. Identification and characterization of pptA: a gene involved in the phase-variable expression of phosphorylcholine on pili of Neisseria meningitidis. Infect Immun 71, 6892-8 (2003).

11.	Gotschlich, E. C. Genetic locus for the biosynthesis of the variable portion of Neisseria gonorrhoeae lipooligosaccharide. J Exp Med 180, 2181-90 (1994).

12.	Yang, Q. L. & Gotschlich, E. C. Variation of gonococcal lipooligosaccharide structure is due to alterations in poly-G tracts in lgt genes encoding glycosyl transferases. J Exp Med 183, 323-7 (1996).

13.	Banerjee, A. et al. Identification of the gene (lgtG) encoding the lipooligosaccharide beta chain synthesizing glucosyl transferase from Neisseria gonorrhoeae. Proc Natl Acad Sci U S A 95, 10872-7 (1998).

14.	Hammerschmidt, S. et al. Capsule phase variation in Neisseria meningitidis serogroup B by slipped-strand mispairing in the polysialyltransferase gene (siaD): correlation with bacterial invasion and the outbreak of meningococcal disease. Mol Microbiol 20, 1211-20 (1996).

15.	Chen, C. J., Elkins, C. & Sparling, P. F. Phase variation of hemoglobin utilization in Neisseria gonorrhoeae. Infect Immun 66, 987-93 (1998).

16.	Richardson, A. R. & Stojiljkovic, I. HmbR, a hemoglobin-binding outer membrane protein of Neisseria meningitidis, undergoes phase variation. J Bacteriol 181, 2067-74 (1999).

17.	Carson, S. D., Stone, B., Beucher, M., Fu, J. & Sparling, P. F. Phase variation of the gonococcal siderophore receptor FetA. Mol Microbiol 36, 585-93 (2000).

18.	Martin, P. et al. Experimentally revised repertoire of putative contingency loci in Neisseria meningitidis strain MC58: evidence for a novel mechanism of phase variation. Mol Microbiol 50, 245-57 (2003).
